# Supplementary material for: Structural and functional evaluation of de novo-designed, two-component nanoparticle carriers for HIV Env trimer immunogens
Source: PLoS Pathog. 2020 Aug 11;16(8):e1008665. doi: 10.1371/journal.ppat.1008665 (PMC7418955; doi:10.1371/journal.ppat.1008665)
Supplement: S1 Table — (DOCX) [file ppat.1008665.s001.docx]

| Env trimers | BG505-SOSIP.v5.2(7S) | MKRGLCCVLLLCGAVFVSPSQEIHARFRRGARAENLWVTVYYGVPVWKDAETTLFCASDAKAYETKKHNVWATHCCVPTDPNPQEIHLENVTEEFNMWKNNMVEQMHTDIISLWDQSLKPCVKLTPLCVTLQCTNVTNNITDDMRGELKNCSFNMTTELRDKKQKVYSLFYRLDVVQINENQGNRSNNSNKEYRLINCNTSAITQACPKVSFEPIPIHYCAPAGFAILKCKDKKFNGTGPCTNVSTVQCTHGIKPVVSTQLLLNGSLAEEEVIIRSENITNNAKNILVQLNESVQINCTRPNNNTVKSIRIGPGQWFYYTGDIIGDIRQAHCNVSKATWNETLGKVVKQLRKHFGNNTIIRFANSSGGDLEVTTHSFNCGGEFFYCNTSGLFNSTWISNTSVQGSNSTGSNDSITLPCRIKQIINMWQRIGQAMYAPPIQGVIRCVSNITGLILTRDGGSTNSTTETFRPGGGDMRDNWRSELYKYKVVKIEPLGVAPTRCKRRVVGRRRRRRAVGIGAVSLGFLGAAGSTMGAASMTLTVQARNLLSGIVQQQSNLLRAPECQQHLLKDTHWGIKQLQARVLAVEHYLRDQQLLGIWGCSGKLICCTNVPWNSSWSNRNLSEIWDNMTWLQWDKEISNYTQIIYGLLEESQNQQEKNEQDLLELD |
| --- | --- | --- |
|  | ConM-SOSIP.v7 | MDAMKRGLCCVLLLCGAVFVSPSQEIHARFRRGARAENLWVTVYYGVPVWKDAETTLFCASDAKAYDTEKRNVWATHCCVPTDPNPQEIVLENVTENFNMWKNNMVEQMHTDIISLWDQSLKPCVKLTPLCVTLNCTDVNATNNTTNNEEIKNCSFNITTELRDKKKKVYALFYKLDVVPIDDNNSYRLINCNTSAITQACPKVSFEPIPIHYCAPAGFAILKCNDKKFNGTGPCKNVSTVQCTHGIKPVVSTQLLLNGSLAEEEIIIRSENITNNAKTIIVQLNESVEINCTRPNNNTRKSIRIGPGQWFYATGDIIGDIRQAHCNISRTKWNKTLQQVAKKLREHFNKTIIFNPSSGGDLEITTHSFNCGGEFFYCNTSELFNSTWNGTNNTITLPCRIKQIINMWQRVGQAMYAPPIEGKIRCTSNITGLLLTRDGGNNNTETFRPGGGDMRDNWRSELYKYKVVKIEPLGVAPTRCKRRVVERRRRRRAVGIGAVFLGFLGAAGSTMGAASMTLTVQARNLLSGIVQQQSNLLRAPECQQHLLQLTVWGIKQLQARVLAVERYLKDQQLLGIWGCSGKLICCTNVPWNSSWSNKSQDEIWDNMTWMEWDKEINNYTDIIYSLIEESQNQQEKNEQELLALD |
|  | ConM-SOSIP.v9 | MDAMKRGLCCVLLLCGAVFVSPSQEIHARFRRGARAENLWVTVYYGVPVWKDAETTLFCASDAKAYDTEKRNVWATHCCVPTDPNPQEIVLENVTENFNMWKNNMVEQMHTDIISLWDQSLKPCVKLTPLCVTLNCTDVNATNNTTNNEEIKNCSFNITTELRDKKKKVYALFYKLDVVPIDDNNSYRLINCNTSAITQACPKVSFEPIPIHYCAPAGFAILKCNDKKFNGTGPCKNVSTVQCTHGIKPVVSTQLLLNGSLAEEEIIIRSENITNNAKTIIVQLNESVEINCTRPNNNTRKSIRIGPGQWFYATGDIIGDIRQAHCNISRTKWNKTLQQVAKKLREHFNKTIIFNPSSGGDLEITTHSFNCGGEFFYCNTSELFNSTWNGTNNTITLPCRIKQIINMWQRVGQAMYAPPIEGKIRCTSNITGLLLTRDGGNNNTETFRPGGGDMRDNWRSELYKYKVVKIEPLGVAPTRCKRRVVERRRRRRAVGIGAVFLGFLGAAGSTMGAASMTLTVQARNLLSGIVQQQSNLLRAPECQQHLLQLTVWGIKQLQARVLAVERYLKDQQLLGIWGCSGKLICCTNVPWNSSWSNKSQDEIWDNMTWMEWDKEINNYTDIIYSLIEESQNQQEKNEQELLALD |
| T33_dn2 | T33_dn2A | MGNLAEKMYKAGNAMYRKGQYTIAIIAYTLALLKDPNNAEAWYNLGNAAYKKGEYDEAIEAYQKALELDPNNAEAWYNLGNAYYKQGDYDEAIEYYKKALRLDPRNVDAIENLIEAEEKQG |
|  | T33_dn2B | MEEAELAYLLGELAYKLGEYRIAIRAYRIALKRDPNNAEAWYNLGNAYYKQGDYREAIRYYLRALKLDPENAEAWYNLGNALYKQGKYDLAIIAYQAALEEDPNNAEAKQNLGNAKQKQGLEHHHHHH |
|  | BG505-SOSIP-T33_dn2A | MKRGLCCVLLLCGAVFVSPSQEIHARFRRGARAENLWVTVYYGVPVWKDAETTLFCASDAKAYETKKHNVWATHCCVPTDPNPQEIHLENVTEEFNMWKNNMVEQMHTDIISLWDQSLKPCVKLTPLCVTLQCTNVTNNITDDMRGELKNCSFNMTTELRDKKQKVYSLFYRLDVVQINENQGNRSNNSNKEYRLINCNTSAITQACPKVSFEPIPIHYCAPAGFAILKCKDKKFNGTGPCTNVSTVQCTHGIKPVVSTQLLLNGSLAEEEVIIRSENITNNAKNILVQLNESVQINCTRPNNNTVKSIRIGPGQWFYYTGDIIGDIRQAHCNVSKATWNETLGKVVKQLRKHFGNNTIIRFANSSGGDLEVTTHSFNCGGEFFYCNTSGLFNSTWISNTSVQGSNSTGSNDSITLPCRIKQIINMWQRIGQAMYAPPIQGVIRCVSNITGLILTRDGGSTNSTTETFRPGGGDMRDNWRSELYKYKVVKIEPLGVAPTRCKRRVVGRRRRRRAVGIGAVSLGFLGAAGSTMGAASMTLTVQARNLLSGIVQQQSNLLRAPECQQHLLKDTHWGIKQLQARVLAVEHYLRDQQLLGIWGCSGKLICCTNVPWNSSWSNRNLSEIWDNMTWLQWDKEISNYTQIIYGLLEESQNQQEKNEQDLLELDKWASLWGSMGNLAEKMYKAGNAMYRKGQYTIAIIAYTLALLKDPNNAEAWYNLGNAAYKKGEYDEAIEAYQKALELDPNNAEAWYNLGNAYYKQGDYDEAIEYYKKALRLDPRNVDAIENLIEAEEKQGAS |
|  | ConM-SOSIP-T33_dn2A | MDAMKRGLCCVLLLCGAVFVSPSQEIHARFRRGARAENLWVTVYYGVPVWKDAETTLFCASDAKAYDTEKRNVWATHCCVPTDPNPQEIVLENVTENFNMWKNNMVEQMHTDIISLWDQSLKPCVKLTPLCVTLNCTDVNATNNTTNNEEIKNCSFNITTELRDKKKKVYALFYKLDVVPIDDNNSYRLINCNTSAITQACPKVSFEPIPIHYCAPAGFAILKCNDKKFNGTGPCKNVSTVQCTHGIKPVVSTQLLLNGSLAEEEIIIRSENITNNAKTIIVQLNESVEINCTRPNNNTRKSIRIGPGQWFYATGDIIGDIRQAHCNISRTKWNKTLQQVAKKLREHFNKTIIFNPSSGGDLEITTHSFNCGGEFFYCNTSELFNSTWNGTNNTITLPCRIKQIINMWQRVGQAMYAPPIEGKIRCTSNITGLLLTRDGGNNNTETFRPGGGDMRDNWRSELYKYKVVKIEPLGVAPTRCKRRVVERRRRRRAVGIGAVFLGFLGAAGSTMGAASMTLTVQARNLLSGIVQQQSNLLRAPECQQHLLQLTVWGIKQLQARVLAVERYLKDQQLLGIWGCSGKLICCTNVPWNSSWSNKSQDEIWDNMTWMEWDKEINNYTDIIYSLIEESQNQQEKNEQELLALDKWASLWGSMGNLAEKMYKAGNAMYRKGQYTIAIIAYTLALLKDPNNAEAWYNLGNAAYKKGEYDEAIEAYQKALELDPNNAEAWYNLGNAYYKQGDYDEAIEYYKKALRLDPRNVDAIENLIEAEEKQGAS |
| T33_dn5 | T33_dn5A | MGNSAEAMYKMGNAAYKQGDYILAIIAYLLALEKDPNNAEAWYNLGNAAYKQGDYDEAIEYYQKALELDPNNAEAWYNLGNAYYKQGDYDEAIEYYEKALELDPNNAEALKNLLEAIAEQD |
|  | T33_dn5B | MGHHHHTDPLAVILYIAILKAEKSIARAKAAEALGKIGDERAVEPLIKALKDEDALVRAAAADALGQIGDERAVEPLIKALKDEEGLVRASAAIALGQIGDERAVQPLIKALTDERDLVRVAAAVALGRIGDEKAVRPLIIVLKDEEGEVREAAAIALGSIGGERVRAAMEKLAERGTGFARKVAVNYLETHKLEHHHHHH |
|  | BG505-SOSIP-T33_dn5A | MKRGLCCVLLLCGAVFVSPSQEIHARFRRGARAENLWVTVYYGVPVWKDAETTLFCASDAKAYETKKHNVWATHCCVPTDPNPQEIHLENVTEEFNMWKNNMVEQMHTDIISLWDQSLKPCVKLTPLCVTLQCTNVTNNITDDMRGELKNCSFNMTTELRDKKQKVYSLFYRLDVVQINENQGNRSNNSNKEYRLINCNTSAITQACPKVSFEPIPIHYCAPAGFAILKCKDKKFNGTGPCTNVSTVQCTHGIKPVVSTQLLLNGSLAEEEVIIRSENITNNAKNILVQLNESVQINCTRPNNNTVKSIRIGPGQWFYYTGDIIGDIRQAHCNVSKATWNETLGKVVKQLRKHFGNNTIIRFANSSGGDLEVTTHSFNCGGEFFYCNTSGLFNSTWISNTSVQGSNSTGSNDSITLPCRIKQIINMWQRIGQAMYAPPIQGVIRCVSNITGLILTRDGGSTNSTTETFRPGGGDMRDNWRSELYKYKVVKIEPLGVAPTRCKRRVVGRRRRRRAVGIGAVSLGFLGAAGSTMGAASMTLTVQARNLLSGIVQQQSNLLRAPECQQHLLKDTHWGIKQLQARVLAVEHYLRDQQLLGIWGCSGKLICCTNVPWNSSWSNRNLSEIWDNMTWLQWDKEISNYTQIIYGLLEESQNQQEKNEQDLLELDKWASLWGSMGNSAEAMYKMGNAAYKQGDYILAIIAYLLALEKDPNNAEAWYNLGNAAYKQGDYDEAIEYYQKALELDPNNAEAWYNLGNAYYKQGDYDEAIEYYEKALELDPNNAEALKNLLEAIAEQD |
| T33_dn10 | T33_dn10A | MGEEAELAYLLGELAYKLGEYRIAIRAYRIALKRDPNNAEAWYNLGNAYYKQGDYDEAIEYYQKALELDPNNAEAWYNLGNAYYKQGDYDEAIEYYEKALELDPENLEALQNLLNAMDKQG |
|  | T33_dn10B | MIEEVVAEMIDILAESSKKSIEELARAADNKTTEKAVAEAIEEIARLATAAIQLIEALAKNLASEEFMARAISAIAELAKKAIEAIYRLADNHTTDTFMARAIAAIANLAVTAILAIAALASNHTTEEFMARAISAIAELAKKAIEAIYRLADNHTTDKFMAAAIEAIALLATLAILAIALLASNHTTEKFMARAIMAIAILAAKAIEAIYRLADNHTSPTYIEKAIEAIEKIARKAIKAIEMLAKNITTEEYKEKAKKIIDIIRKLAKMAIKKLEDNRTLEHHHHHH |
|  | BG505-SOSIP-T33_dn10A | MKRGLCCVLLLCGAVFVSPSQEIHARFRRGARAENLWVTVYYGVPVWKDAETTLFCASDAKAYETKKHNVWATHCCVPTDPNPQEIHLENVTEEFNMWKNNMVEQMHTDIISLWDQSLKPCVKLTPLCVTLQCTNVTNNITDDMRGELKNCSFNMTTELRDKKQKVYSLFYRLDVVQINENQGNRSNNSNKEYRLINCNTSAITQACPKVSFEPIPIHYCAPAGFAILKCKDKKFNGTGPCTNVSTVQCTHGIKPVVSTQLLLNGSLAEEEVIIRSENITNNAKNILVQLNESVQINCTRPNNNTVKSIRIGPGQWFYYTGDIIGDIRQAHCNVSKATWNETLGKVVKQLRKHFGNNTIIRFANSSGGDLEVTTHSFNCGGEFFYCNTSGLFNSTWISNTSVQGSNSTGSNDSITLPCRIKQIINMWQRIGQAMYAPPIQGVIRCVSNITGLILTRDGGSTNSTTETFRPGGGDMRDNWRSELYKYKVVKIEPLGVAPTRCKRRVVGRRRRRRAVGIGAVSLGFLGAAGSTMGAASMTLTVQARNLLSGIVQQQSNLLRAPECQQHLLKDTHWGIKQLQARVLAVEHYLRDQQLLGIWGCSGKLICCTNVPWNSSWSNRNLSEIWDNMTWLQWDKEISNYTQIIYGLLEESQNQQEKNEQGSGSGSGSGGEEAELAYLLGELAYKLGEYRIAIRAYRIALKRDPNNAEAWYNLGNAYYKQGDYDEAIEYYQKALELDPNNAEAWYNLGNAYYKQGDYDEAIEYYEKALELDPENLEALQNLLNAMDKQG |
| O43_dn18 | O43_dn18B | MEEAELAYLLGELAYKLGEYRIAIRAYRIALKRDPNNAEAWYNLGNAYYKQGDYDEAIEYYQKALELDPNNAEAWYNLGNAYYKQGDYDEAIEYYQKALELDPSNLDAAVNLGAATMLTS |
|  | O43-dn18A | MDRCEELARRIAEVVERAKRAGTSEDEIAESVARVISLVIRALKLSGSSYEVICECVARIVAEIVEALKRSGTSAVEIAKIVARVISEVIRTLKESGSSYEVICECVARIVAEIVEALKRSGTSAAIIALIVALVISEVIRTLKESGSSFEVILECVIRIVLEIIEALKRSGTSEQDVMLIVMAVLLVVLATLQLSGSHHHHHH |
|  | BG505-SOSIP-O43_dn18B | MKRGLCCVLLLCGAVFVSPSQEIHARFRRGARAENLWVTVYYGVPVWKDAETTLFCASDAKAYETKKHNVWATHCCVPTDPNPQEIHLENVTEEFNMWKNNMVEQMHTDIISLWDQSLKPCVKLTPLCVTLQCTNVTNNITDDMRGELKNCSFNMTTELRDKKQKVYSLFYRLDVVQINENQGNRSNNSNKEYRLINCNTSAITQACPKVSFEPIPIHYCAPAGFAILKCKDKKFNGTGPCTNVSTVQCTHGIKPVVSTQLLLNGSLAEEEVIIRSENITNNAKNILVQLNESVQINCTRPNNNTVKSIRIGPGQWFYYTGDIIGDIRQAHCNVSKATWNETLGKVVKQLRKHFGNNTIIRFANSSGGDLEVTTHSFNCGGEFFYCNTSGLFNSTWISNTSVQGSNSTGSNDSITLPCRIKQIINMWQRIGQAMYAPPIQGVIRCVSNITGLILTRDGGSTNSTTETFRPGGGDMRDNWRSELYKYKVVKIEPLGVAPTRCKRRVVGRRRRRRAVGIGAVSLGFLGAAGSTMGAASMTLTVQARNLLSGIVQQQSNLLRAPECQQHLLKDTHWGIKQLQARVLAVEHYLRDQQLLGIWGCSGKLICCTNVPWNSSWSNRNLSEIWDNMTWLQWDKEISNYTQIIYGLLEESQNQQEKNEQGSGSGSGSGSEEAELAYLLGELAYKLGEYRIAIRAYRIALKRDPNNAEAWYNLGNAYYKQGDYDEAIEYYQKALELDPNNAEAWYNLGNAYYKQGDYDEAIEYYQKALELDPSNLDAAVNLGAATMLTS |
| I53_dn5 | I53-dn5B | MEEAELAYLLGELAYKLGEYRIAIRAYRIALKRDPNNAEAWYNLGNAYYKQGRYREAIEYYQKALELDPNNAEAWYNLGNAYYERGEYEEAIEYYRKALRLDPNNADAMQNLLNAKMREELE |
|  | I53-dn5A | MGKYDGSKLRIGILHARWNAEIILALVLGALKRLQEFGVKRENIIIETVPGSFELPYGSKLFVEKQKRLGKPLDAIIPIGVLIKGSTMHFEYICDSTTHQLMKLNFELGIPVIFGVLTCLTDEQAEARAGLIEGKMHNHGEDWGAAAVEMATKFNLEHHHHHH |
|  | BG505-SOSIP-I53_dn5B | MKRGLCCVLLLCGAVFVSPSQEIHARFRRGARAENLWVTVYYGVPVWKDAETTLFCASDAKAYETKKHNVWATHCCVPTDPNPQEIHLENVTEEFNMWKNNMVEQMHTDIISLWDQSLKPCVKLTPLCVTLQCTNVTNNITDDMRGELKNCSFNMTTELRDKKQKVYSLFYRLDVVQINENQGNRSNNSNKEYRLINCNTSAITQACPKVSFEPIPIHYCAPAGFAILKCKDKKFNGTGPCTNVSTVQCTHGIKPVVSTQLLLNGSLAEEEVIIRSENITNNAKNILVQLNESVQINCTRPNNNTVKSIRIGPGQWFYYTGDIIGDIRQAHCNVSKATWNETLGKVVKQLRKHFGNNTIIRFANSSGGDLEVTTHSFNCGGEFFYCNTSGLFNSTWISNTSVQGSNSTGSNDSITLPCRIKQIINMWQRIGQAMYAPPIQGVIRCVSNITGLILTRDGGSTNSTTETFRPGGGDMRDNWRSELYKYKVVKIEPLGVAPTRCKRRVVGRRRRRRAVGIGAVSLGFLGAAGSTMGAASMTLTVQARNLLSGIVQQQSNLLRAPECQQHLLKDTHWGIKQLQARVLAVEHYLRDQQLLGIWGCSGKLICCTNVPWNSSWSNRNLSEIWDNMTWLQWDKEISNYTQIIYGLLEESQNQQEKNEQGSGSGSGSGGEEAELAYLLGELAYKLGEYRIAIRAYRIALKRDPNNAEAWYNLGNAYYKQGRYREAIEYYQKALELDPNNAEAWYNLGNAYYERGEYEEAIEYYRKALRLDPNNADAMQNLLNAKMREELEAS |
