# Supplementary material for: Structural and functional evaluation of de novo-designed, two-component nanoparticle carriers for HIV Env trimer immunogens
Source: PLoS Pathog. 2020 Aug 11;16(8):e1008665. doi: 10.1371/journal.ppat.1008665 (PMC7418955; doi:10.1371/journal.ppat.1008665)
Supplement: S2 Table — (DOCX) [file ppat.1008665.s002.docx]

|  | **Mutations** | **MD39** | **v5.2** | **v5.2(7S)** |
| --- | --- | --- | --- | --- |
| **Stabilizing**  **mutations** | A501C |  |  |  |
|  | T605C |  |  |  |
|  | I559P |  |  |  |
|  | E64K |  |  |  |
|  | A73C |  |  |  |
|  | A316W |  |  |  |
|  | A561C |  |  |  |
|  | M271I |  |  |  |
|  | A319Y |  |  |  |
|  | R585H |  |  |  |
|  | L568D |  |  |  |
|  | V570H |  |  |  |
|  | R304V |  |  |  |
|  | F519S |  |  |  |
|  | T106E |  |  |  |
|  | A561P |  |  |  |
|  | F223W |  |  |  |
|  | N363Q |  |  |  |
| **Glycan knock-in mutations** | P240T |  |  |  |
|  | S241N |  |  |  |
|  | F288L |  |  |  |
|  | T290E |  |  |  |
|  | P291S |  |  |  |
