# Supplementary material for: Structural and functional evaluation of de novo-designed, two-component nanoparticle carriers for HIV Env trimer immunogens
Source: PLoS Pathog. 2020 Aug 11;16(8):e1008665. doi: 10.1371/journal.ppat.1008665 (PMC7418955; doi:10.1371/journal.ppat.1008665)
Supplement: S3 Table — (DOCX) [file ppat.1008665.s003.docx]

|  | **BG505-SOSIP-T33_dn10** | **BG505-SOSIP-I53_dn5** |
| --- | --- | --- |
| **Microscope** | Talos Arctica | Talos Arctica |
| **Voltage (kV)** | 200 | 200 |
| **Detector** | Gatan K2 Summit | Gatan K2 Summit |
| **Recording mode** | Counting | Counting |
| **Magnification** | 36,000 X | 36,000 X |
| **Movie micrograph pixel size** | 1.15 | 1.15 |
| **Dose rate (e^−^/Å^2^/s)** | 4.39 | 4.39 |
| **No. of frames per movie micrograph** | 45 | 45 |
| **Frame exposure time (ms)** | 250 | 250 |
| **Movie micrograph exposure time (s)** | 11.25 | 11.25 |
| **Total dose (e^−^/Å^2^)** | 49.39 | 49.39 |
| **Under focus range (µm)** | 0.6 – 2.0 | 0.8 – 2.0 |
| **Number of movie micrographs** | 798 | 2,999 |
