# Supplementary material for: Structural and functional evaluation of de novo-designed, two-component nanoparticle carriers for HIV Env trimer immunogens
Source: PLoS Pathog. 2020 Aug 11;16(8):e1008665. doi: 10.1371/journal.ppat.1008665 (PMC7418955; doi:10.1371/journal.ppat.1008665)
Supplement: S4 Table — (DOCX) [file ppat.1008665.s004.docx]

|  | **T33_dn10 core** | **BG505-SOSIP Trimer** |
| --- | --- | --- |
| **PDB** | 6VFK | 6VFL |
| **Residues** | 4764 | 1812 |
| **Amino-acids** | 4764 | 1710 |
| **Carbohydrates** | 0 | 102 |
| **RMSD Bonds** | 0.021 | 0.019 |
| **RMSD Angles** | 1.411 | 1.605 |
| **Ramachandran** |  |  |
| **Outliers (%)** | 0.00 | 0.00 |
| **Allowed (%)** | 1.27 | 1.43 |
| **Favored (%)** | 98.73 | 98.57 |
| **Rotamer outliers** | 0.00 | 0.20 |
| **Clash score** | 0.93 | 1.09 |
| **Molprobity score** | 0.78 | 0.81 |
| **EMRinger score** | 0.80 | 2.59 |
