# Supplementary material for: Structural and functional evaluation of de novo-designed, two-component nanoparticle carriers for HIV Env trimer immunogens
Source: PLoS Pathog. 2020 Aug 11;16(8):e1008665. doi: 10.1371/journal.ppat.1008665 (PMC7418955; doi:10.1371/journal.ppat.1008665)
Supplement: S5 Table — (DOCX) [file ppat.1008665.s005.docx]

|  | **Group 1:**  **ConM-SOSIP.v7** | **Group 2:**  **ConM-SOSIP-T33_dn2** |
| --- | --- | --- |
| **Week 2** | 1708 | 1291 |
|  | 523 | 2724 |
|  | 1066 | 3475 |
|  | 1150 | 1104 |
|  | 1974 | 994 |
| **Week 4** | 13093 | 11520 |
|  | 2000 | 14856 |
|  | 2000 | 22091 |
|  | 9416 | 13037 |
|  | 11404 | 17761 |
| **Week 6** | 108854 | 87724 |
|  | 74585 | 76419 |
|  | 35315 | 324551 |
|  | 128281 | 85759 |
|  | 172543 | 104409 |
| **Week 8** | 50271 | 43075 |
|  | 25641 | 30468 |
|  | 17550 | 108355 |
|  | 83114 | 27938 |
|  | 49718 | 38000 |
| **Week 12** | 20066 | 15544 |
|  | 14839 | 12784 |
|  | 19127 | 61362 |
|  | 32819 | 14558 |
|  | 163139 | 19501 |
| **Week 22** | 142830 | 107669 |
|  | 180083 | 265958 |
|  | 313246 | 252046 |
|  | 113955 | 253543 |
|  | 938193 | 75207 |
