# Supplementary material for: Structural and functional evaluation of de novo-designed, two-component nanoparticle carriers for HIV Env trimer immunogens
Source: PLoS Pathog. 2020 Aug 11;16(8):e1008665. doi: 10.1371/journal.ppat.1008665 (PMC7418955; doi:10.1371/journal.ppat.1008665)
Supplement: S6 Table — Midpoint neutralization titers (ID50) were determined using TZM-bl neutralization assays and sera samples from different time points (indicated in the top row). Neutralization assays with murine leukemia virus (MLV) were also performed (negative control). Color coding: white = no neutralization (ID50 < 20); yellow = very weak neutralization (20 < ID50 < 100); light orange = moderate neutralization (100 < ID50 < 1000); dark orange = strong neutralization (1000 < ID50 < 10000); red = very strong neutralization (ID50 > 10000). (DOCX) [file ppat.1008665.s006.docx]

|  |  | **Week 4** | | **Week 6** | | **Week 8** | **Week 12** | **Week 16** | **Week 20** | **Week 22** | |
| --- | --- | --- | --- | --- | --- | --- | --- | --- | --- | --- | --- |
|  | Virus | ConM | MLV | ConM | MLV | ConM | ConM | ConM | ConM | ConM | MLV |
| Immunogen | Rabbit ID |  | | | | | | | | | |
| ConM-SOSIP.v7 | 2378 | 117 | 59 | 2776 | 75 | 1874 | 1566 | 1412 | 709 | 8118 | 45 |
|  | 2379 | <20 | 22 | <20 | <20 | 41 | 94 | 203 | 80 | 1703 | <20 |
|  | 2380 | <20 | <20 | 188 | <20 | 71 | 47 | 174 | 55 | 12901 | <20 |
|  | 2381 | <20 | 21 | 3015 | <20 | 1249 | 2228 | 1810 | 1481 | 17335 | 28 |
|  | 2382 | 152 | <20 | 11815 | <20 | 4502 | 1113 | 1177 | 556 | 53063 | <20 |
| ConM-SOSIP-T33_dn2 | 2383 | 97 | <20 | 4833 | <20 | 6192 | 17518 | 1264 | 1252 | 37279 | <20 |
|  | 2384 | 2413 | <20 | 10820 | <20 | 8540 | 6037 | 3697 | 1715 | 33662 | <20 |
|  | 2385 | 1562 | <20 | 17611 | <20 | 35064 | 6745 | 4489 | 3141 | 194798 | <20 |
|  | 2386 | 86 | <20 | 2816 | <20 | 6581 | 502 | 841 | 597 | 9767 | <20 |
|  | 2387 | 106 | <20 | 3395 | <20 | 1601 | 1897 | 4794 | 2195 | 36643 | <20 |

* MLV titers measured only for sera samples from weeks: 4, 6 and 22 (after each immunization)
