# Supplementary material for: Structural and functional evaluation of de novo-designed, two-component nanoparticle carriers for HIV Env trimer immunogens
Source: PLoS Pathog. 2020 Aug 11;16(8):e1008665. doi: 10.1371/journal.ppat.1008665 (PMC7418955; doi:10.1371/journal.ppat.1008665)
Supplement: S7 Table — The name and tier classification for each HIV Env sequence is indicated. Color coding: white = no neutralization (ID50 < 20); yellow = very weak neutralization (20 < ID50 < 100); light orange = moderate neutralization (100 < ID50 < 1000); dark orange = strong neutralization (1000 < ID50 < 10000); red = very strong neutralization (ID50 > 10000). Toxicity was observed at 1:20 dilution for all samples highlighted in gray. (DOCX) [file ppat.1008665.s007.docx]

|  |  | **Week 22** | | | | | | | | | |
| --- | --- | --- | --- | --- | --- | --- | --- | --- | --- | --- | --- |
|  | Virus | ConM | 25710-2.43 | TRO.11 | BJOX002000.  03.2 | X1632-S2-B10 | Ce1176_A3 | 246-F3_C10_2 | CH119.10 | Ce703010  217_B6 | CNE55 |
|  | Tier | 1A | 2 | 2 | 2 | 2 | 2 | 2 | 2 | 2 | 2 |
| Immunogen | Rabbit ID |  | | | | | | | | | |
| ConM-SOSIP.v7 | 2378 | 8118 | <20 | <20 | <20 | <20 | <20 | <20 | <20 | <20 | <20 |
|  | 2379 | 1703 | <20 | <20 | <20 | <20 | <20 | <20 | <20 | <20 | <20 |
|  | 2380 | 12901 | <20 | <20 | <20 | <20 | <20 | <20 | <20 | <20 | <20 |
|  | 2381 | 17335 | **39** | **41** | **41** | **39** | **40** | **43** | **36** | **34** | **33** |
|  | 2382 | 53063 | <20 | <20 | <20 | <20 | <20 | <20 | <20 | <20 | <20 |
| ConM-SOSIP-T33_dn2 | 2383 | 37279 | <20 | <20 | <20 | <20 | <20 | <20 | **22** | <20 | <20 |
|  | 2384 | 33662 | <20 | <20 | <20 | <20 | <20 | <20 | <20 | <20 | <20 |
|  | 2385 | 194798 | <20 | <20 | <20 | <20 | <20 | <20 | <20 | <20 | <20 |
|  | 2386 | 9767 | <20 | <20 | <20 | <20 | <20 | <20 | <20 | <20 | <20 |
|  | 2387 | 36643 | <20 | <20 | <20 | <20 | <20 | <20 | <20 | <20 | <20 |
| CH103-31 | IC_50_^#^ |  | 0.28 | 0.37 | 8.8 | 0.11 | 0.18 | 0.16 | 1.3 | 0.09 | 0.17 |

# IC_50_ values for CH103-31 are in µg/ml
