# Supplementary material for: Structural and functional evaluation of de novo-designed, two-component nanoparticle carriers for HIV Env trimer immunogens
Source: PLoS Pathog. 2020 Aug 11;16(8):e1008665. doi: 10.1371/journal.ppat.1008665 (PMC7418955; doi:10.1371/journal.ppat.1008665)
Supplement: S8 Table — Color coding: white = no neutralization (ID50 < 20); yellow = very weak neutralization (20 < ID50 < 100); light orange = moderate neutralization (100 < ID50 < 1000); dark orange = strong neutralization (1000 < ID50 < 10000); red = very strong neutralization (ID50 > 10000). (DOCX) [file ppat.1008665.s008.docx]

|  |  | **Week 22** | | | | |
| --- | --- | --- | --- | --- | --- | --- |
|  | Virus | ConM | ConS | SF162 | MN.3 | MW965 |
|  | Tier | 1a | 1b | 1a | 1a | 1a |
| Immunogen | Rabbit ID |  | | | | |
| ConM-SOSIP.v7 | 2378 | 8118 | 476 | 113 | <20 | 30 |
|  | 2379 | 1703 | 174 | <20 | <20 | 945 |
|  | 2380 | 12901 | 422 | <20 | <20 | 43 |
|  | 2381 | 17335 | 1422 | 48 | 34 | 199 |
|  | 2382 | 53063 | 1311 | <20 | 22 | 1062 |
| ConM-SOSIP-T33_dn2 | 2383 | 37279 | 583 | 24 | 41 | 519 |
|  | 2384 | 33662 | 1606 | <20 | 32 | 145 |
|  | 2385 | 194798 | 4804 | 31 | 38 | 72 |
|  | 2386 | 9767 | 493 | 143 | 113 | 393 |
|  | 2387 | 36643 | 305 | 22 | <20 | 72 |
