# Supplementary figures and images for: Structural and functional evaluation of de novo-designed, two-component nanoparticle carriers for HIV Env trimer immunogens
Source: PLoS Pathog. 2020 Aug 11;16(8):e1008665. doi: 10.1371/journal.ppat.1008665 (PMC7418955; doi:10.1371/journal.ppat.1008665)

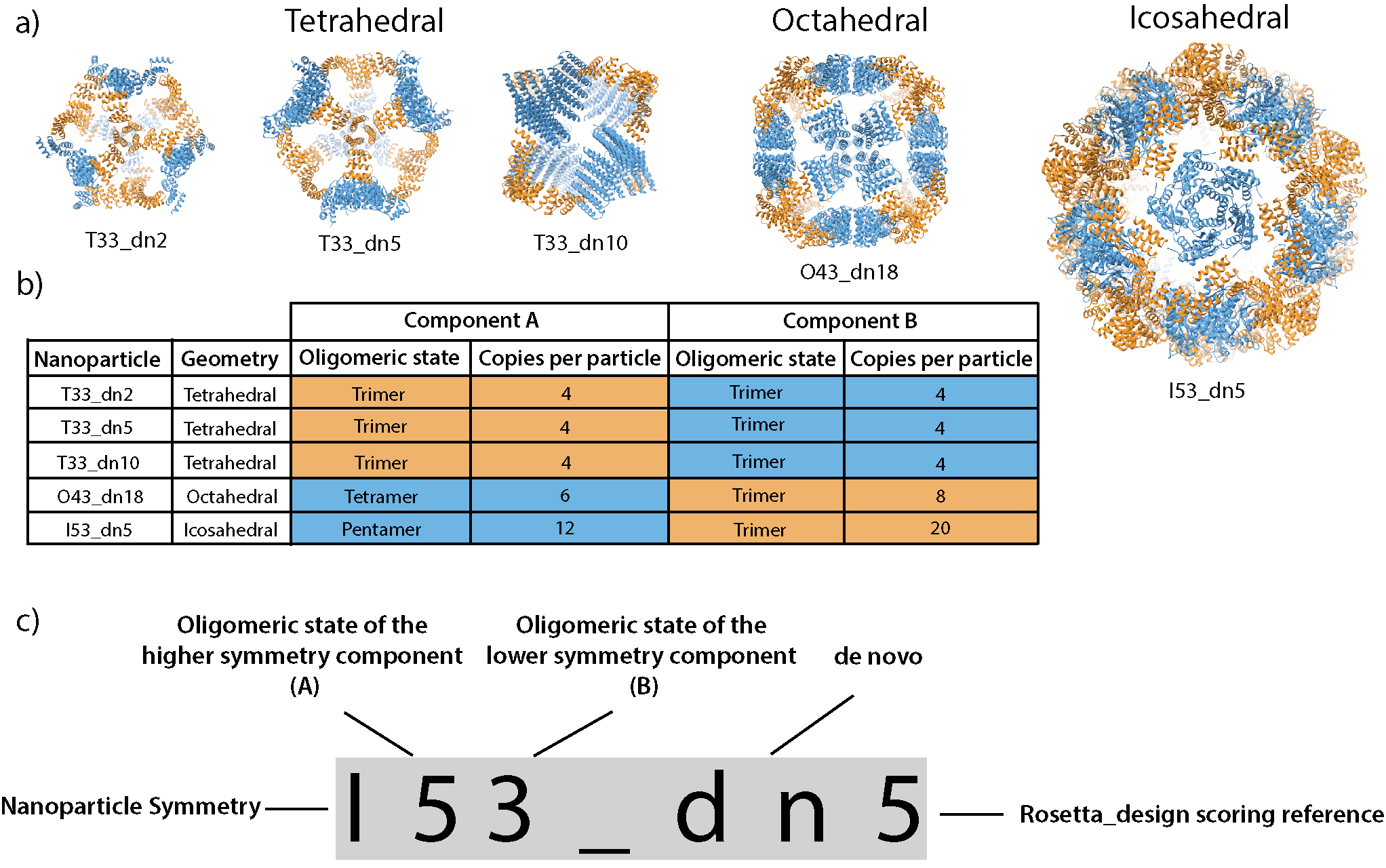

Supplement: S1 Fig — (a) Structural models of nanoparticle candidates derived from Rosetta_design. For clarity, trimeric antigen-bearing component is shown in orange and assembly component in blue. (b) Geometric properties of different nanoparticle candidates. (c) Nanoparticle naming system explained on the example of I53_dn5. (TIF) [file ppat.1008665.s009.tif]

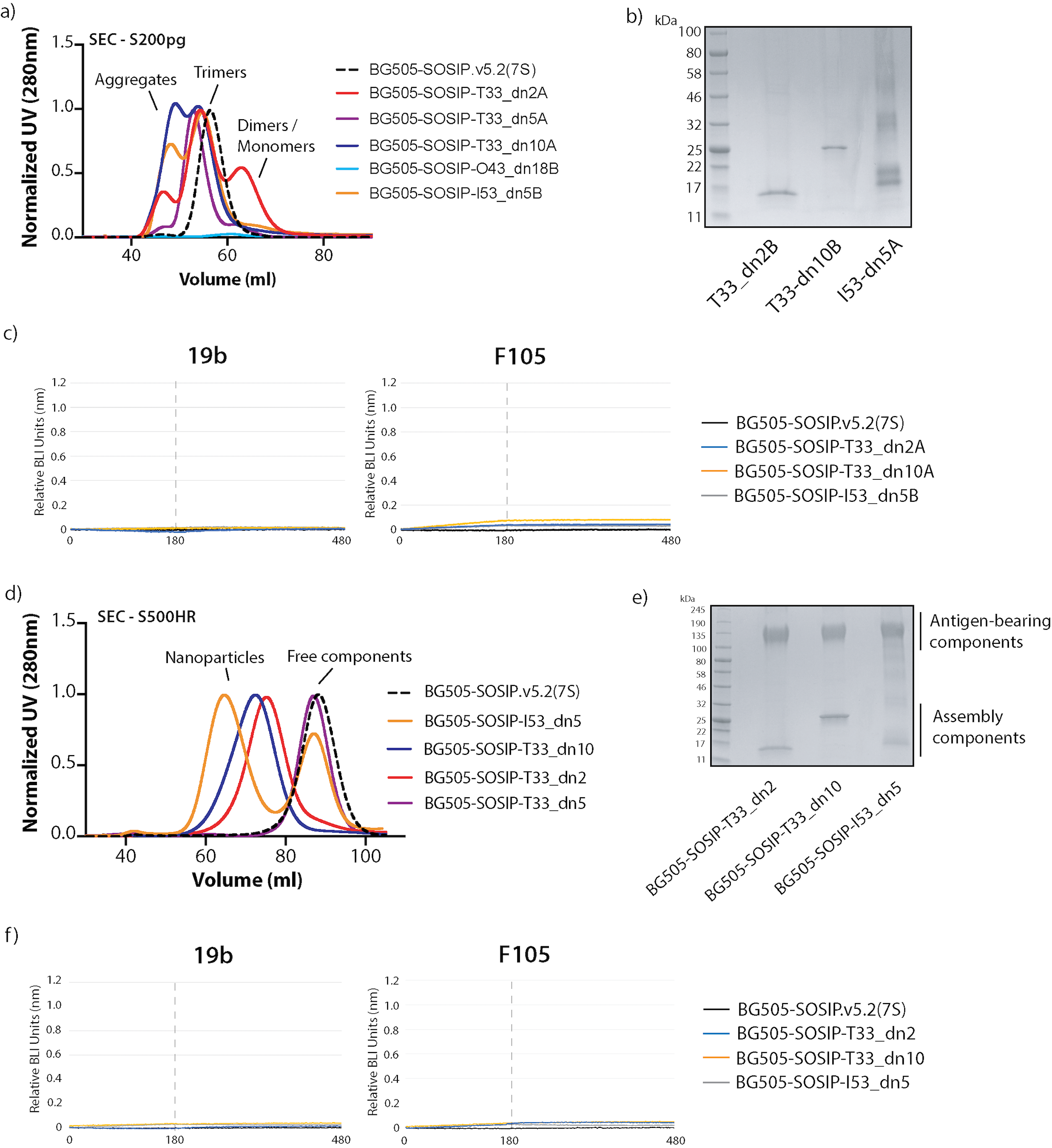

Supplement: S2 Fig — (a) SEC curves of BG505-SOSIP.v5.2(7S) and BG505-SOSIP-fused nanoparticle components. (b) SDS PAGE analysis of the purified assembly component for T33_dn2, T33_dn10 and I53_dn5 nanoparticle systems. (c) Extended data on BLI characterization of the antigenicity of the three antigen-bearing components compared to BG505-SOSIP.v5.2(7S) using 19b and F105 antibodies. (d) SEC purification of different nanoparticle candidates after assembly. (e) SDS PAGE gel of the purified nanoparticles confirming the presence of both, antigen-bearing and assembly components. (f) Extended data on BLI characterization of the antigenicity of the three nanoparticle systems compared to BG505-SOSIP.v5.2(7S) using 19b and F105 antibodies. (TIF) [file ppat.1008665.s010.tif]

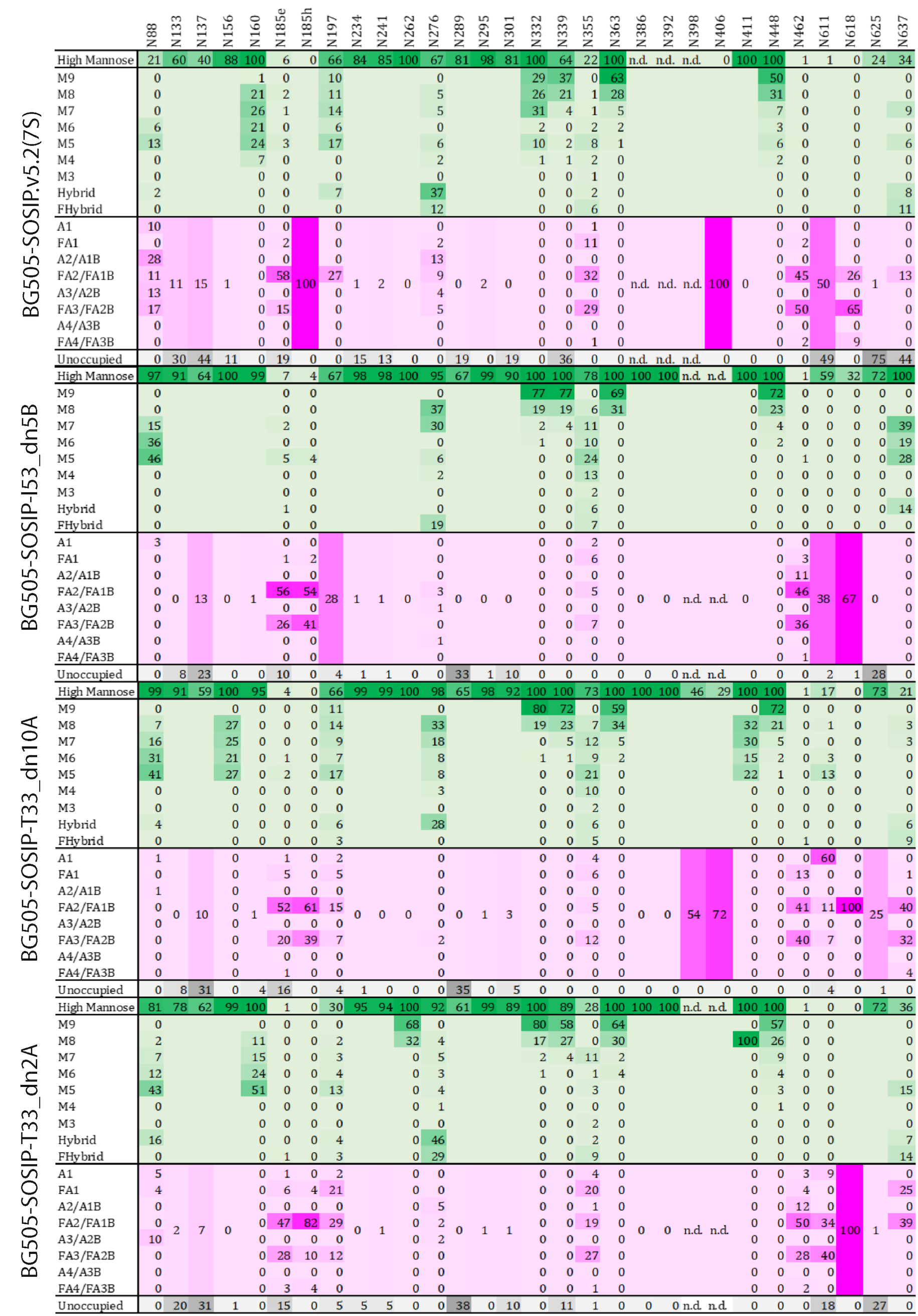

Supplement: S3 Fig — The table shows the glycoforms found at each potential N-linked glycosylation site (PNGS), compositions corresponding to oligomannose/hybrid-type glycans are colored green and fully processed complex type glycans are colored magenta. PNGS with no attached glycan are colored grey. Oligomannose-type glycans are categorized according to the number of mannose residues present, hybrids are categorized according to the presence/absence of fucose and complex-type glycans are categorized according to the number of processed antenna and the presence/absence of fucose. Sites that could only be obtained from low intensity peptides cannot be distinguished into the categories in the table and so are merged to cover all oligomannose/hybrid compositions or complex-type glycans. (TIF) [file ppat.1008665.s011.tif]

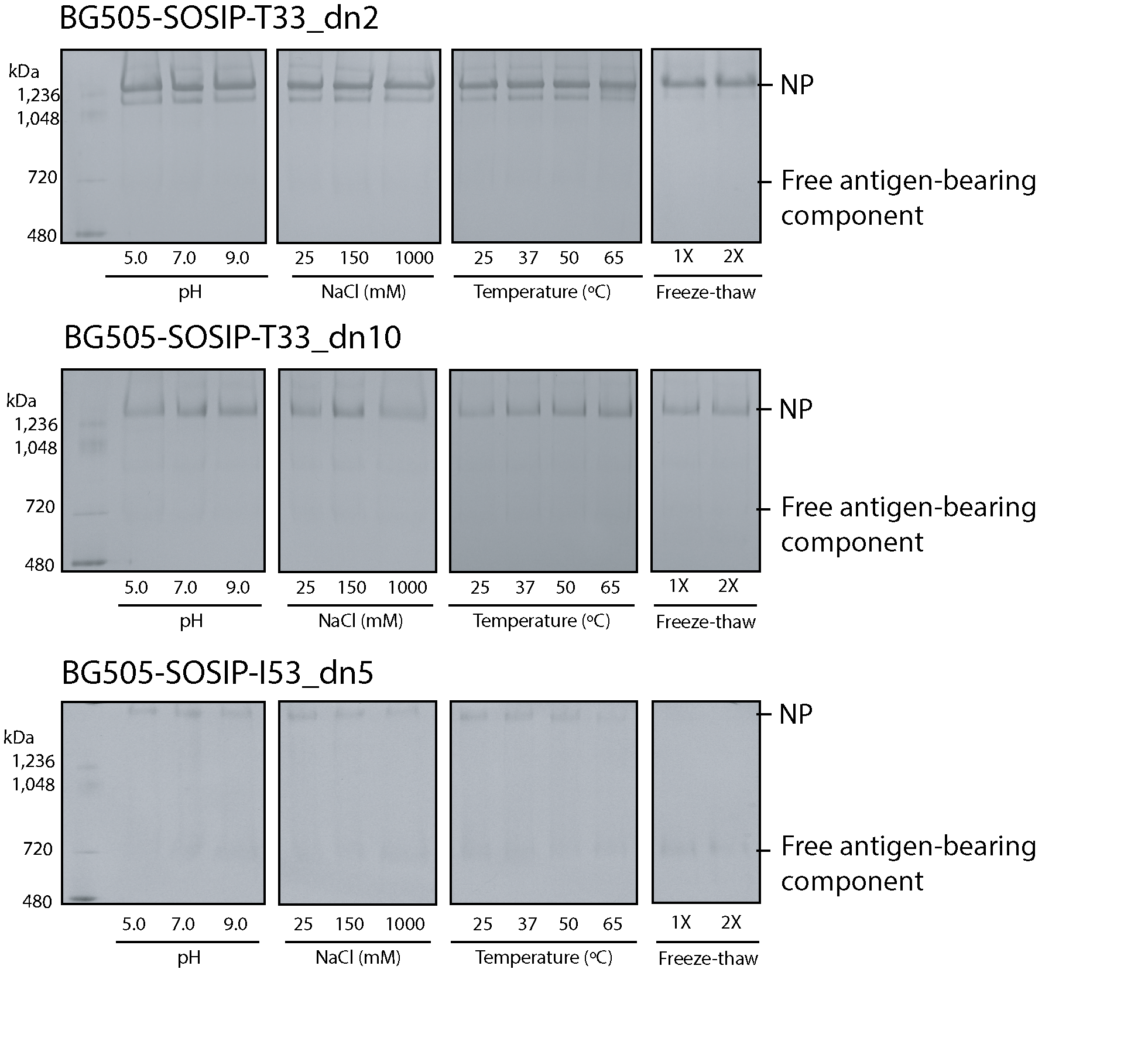

Supplement: S4 Fig — Native PAGE assays were used for evaluation of nanoparticle integrity following the incubation under the specified conditions. (TIF) [file ppat.1008665.s012.tif]

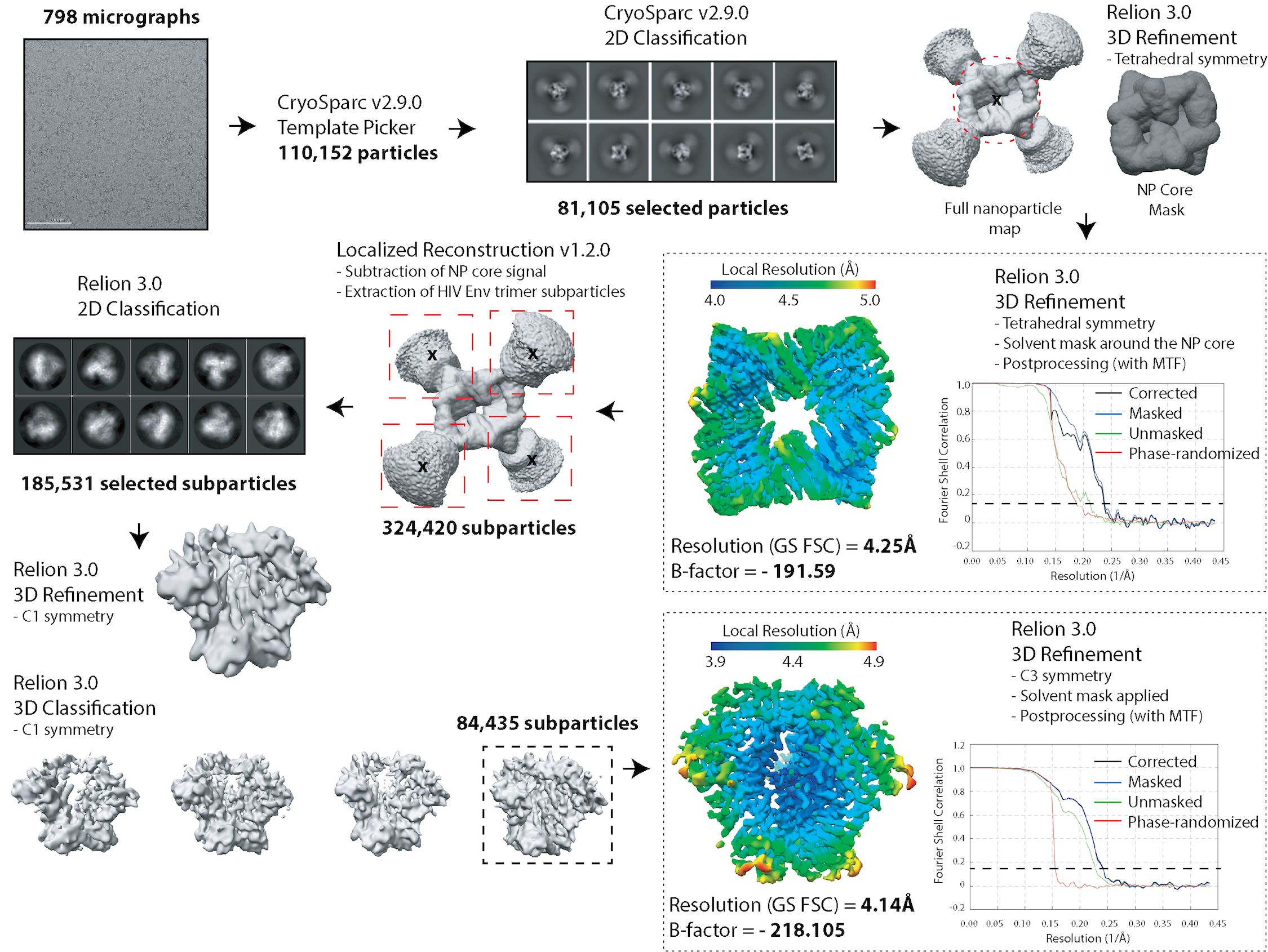

Supplement: S5 Fig — Schematic representation of the data processing workflow with relevant statistics. (TIF) [file ppat.1008665.s013.tif]

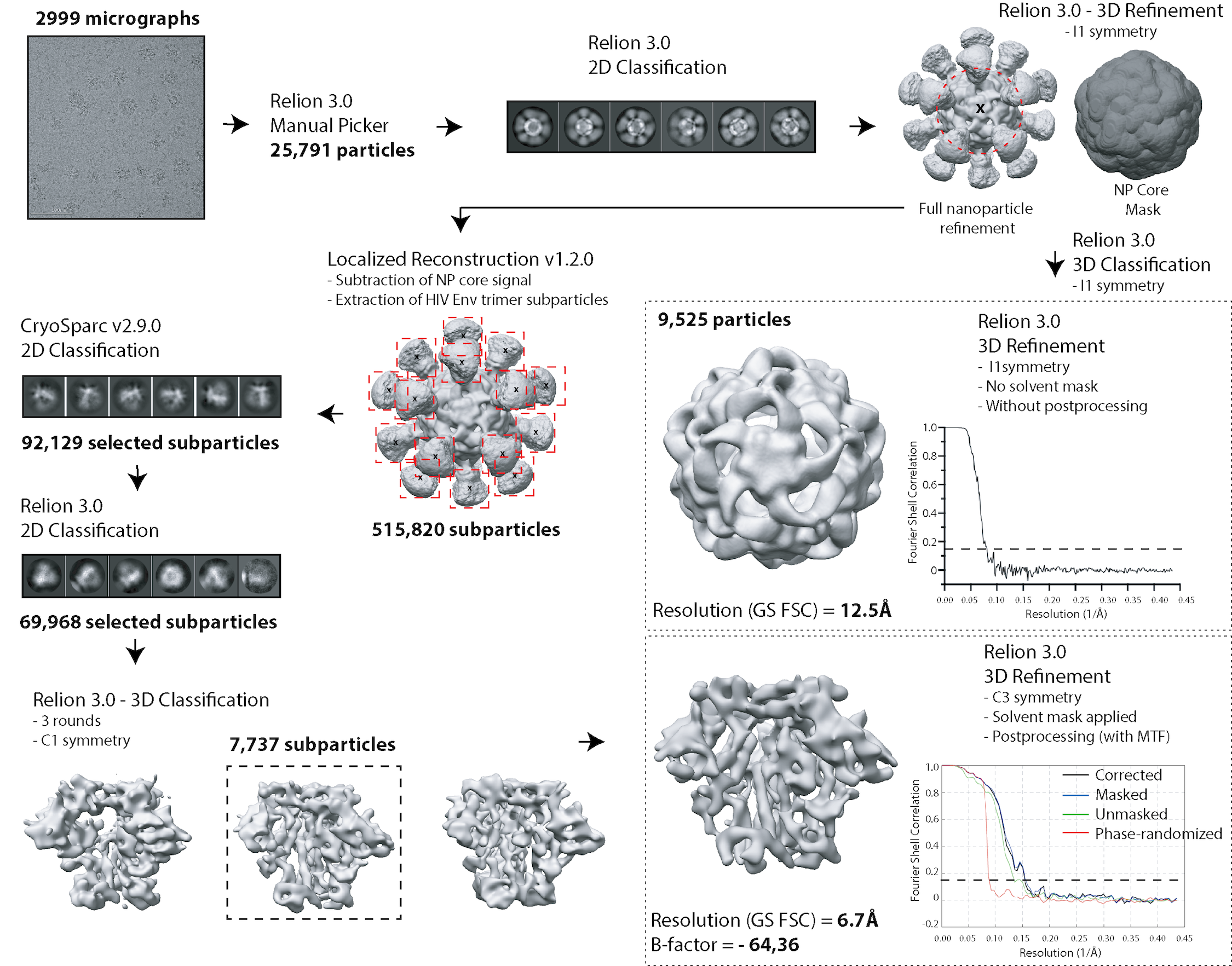

Supplement: S6 Fig — Schematic representation of the data processing workflow with relevant statistics. (TIF) [file ppat.1008665.s014.tif]

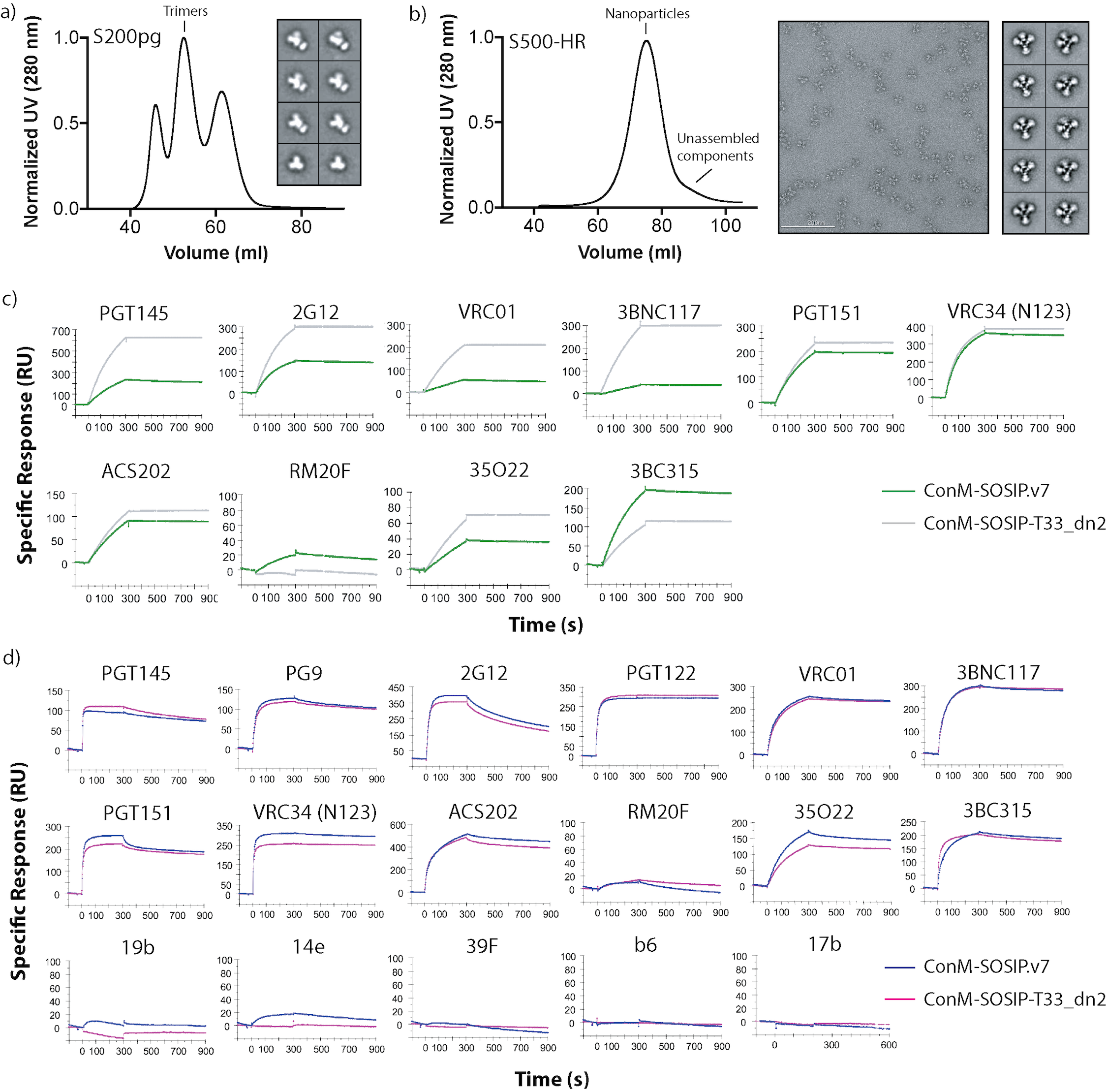

Supplement: S7 Fig — (a) SEC purification of ConM-SOSIP-T33_dn2A and 2D class-averages from negative-stain-EM analysis. (b) SEC purification of assembled ConM-SOSIP-T33_dn2 and NS-EM analysis of the purified nanoparticles (raw micrograph and 2D class averages). (c) SPR-based characterization of the antigenicity of purified nanoparticles with immobilization of monoclonal antibodies (antigens were in the mobile phase). ConM-SOSIP.v7 trimer was used as a reference. In addition to affinity, SPR signal is also a function of antigen size (molecular weight). MW of the ConM-SOSIP-T33_dn2 nanoparticle is ~5.1 times higher than that of soluble ConM-SOSIP.v7 trimer. See methods section for data analysis information. (d) SPR-based characterization of the antigenicity of purified nanoparticles with immobilization of antigens (antibodies were in the mobile phase). ConM-SOSIP.v7 was used as a reference. (TIF) [file ppat.1008665.s015.tif]

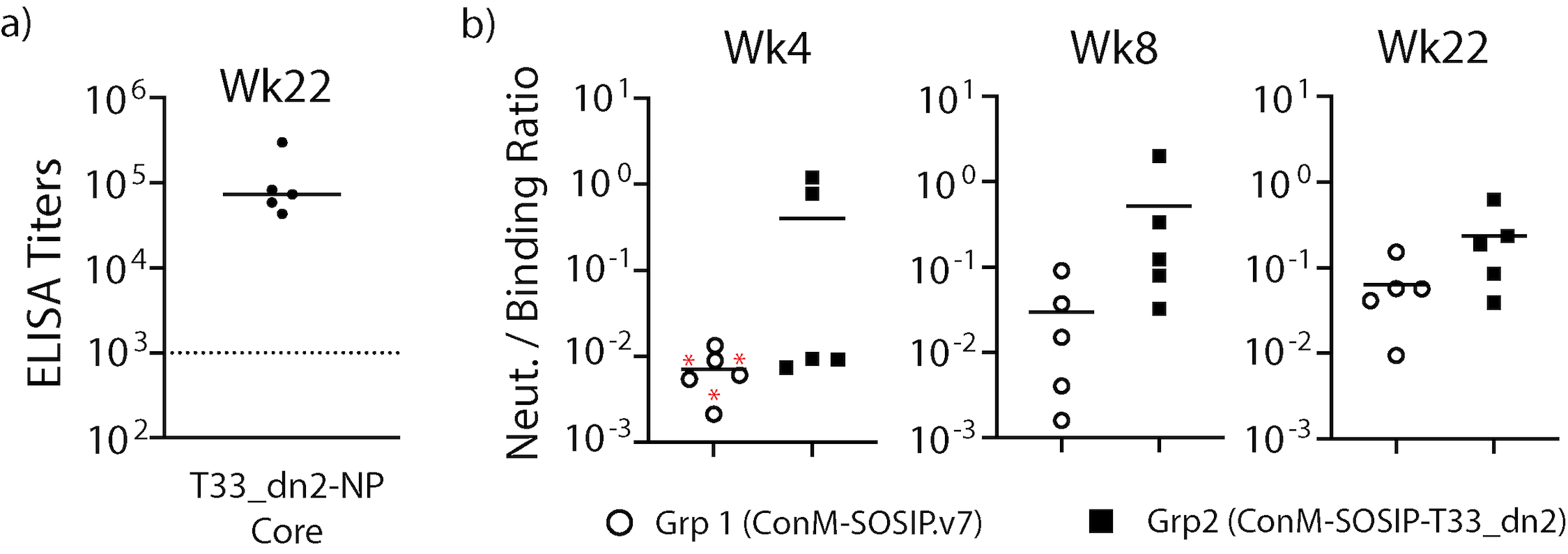

Supplement: S8 Fig — (a) Anti-nanoparticle core response (ELISA binding titers) in individual Group 2 animals, with the mean value indicated by the solid line. The dashed line represents the assay detection limit. (b) Ratios of NAb titers and anti-trimer binding antibody titers in sera from individual animals in Group 1 (open circles) and Group 2 (black squares) were calculated at weeks 4, 8 and 22. Scatter plots are shown with mean values indicated. Red asterisks indicate sera samples where neutralization titers were below the level of detection (1:20 titer). An AUC statistical analysis of the titer ratio values for Group 1 versus Group 2 as a function of time results in p = 0.016. (TIF) [file ppat.1008665.s016.tif]

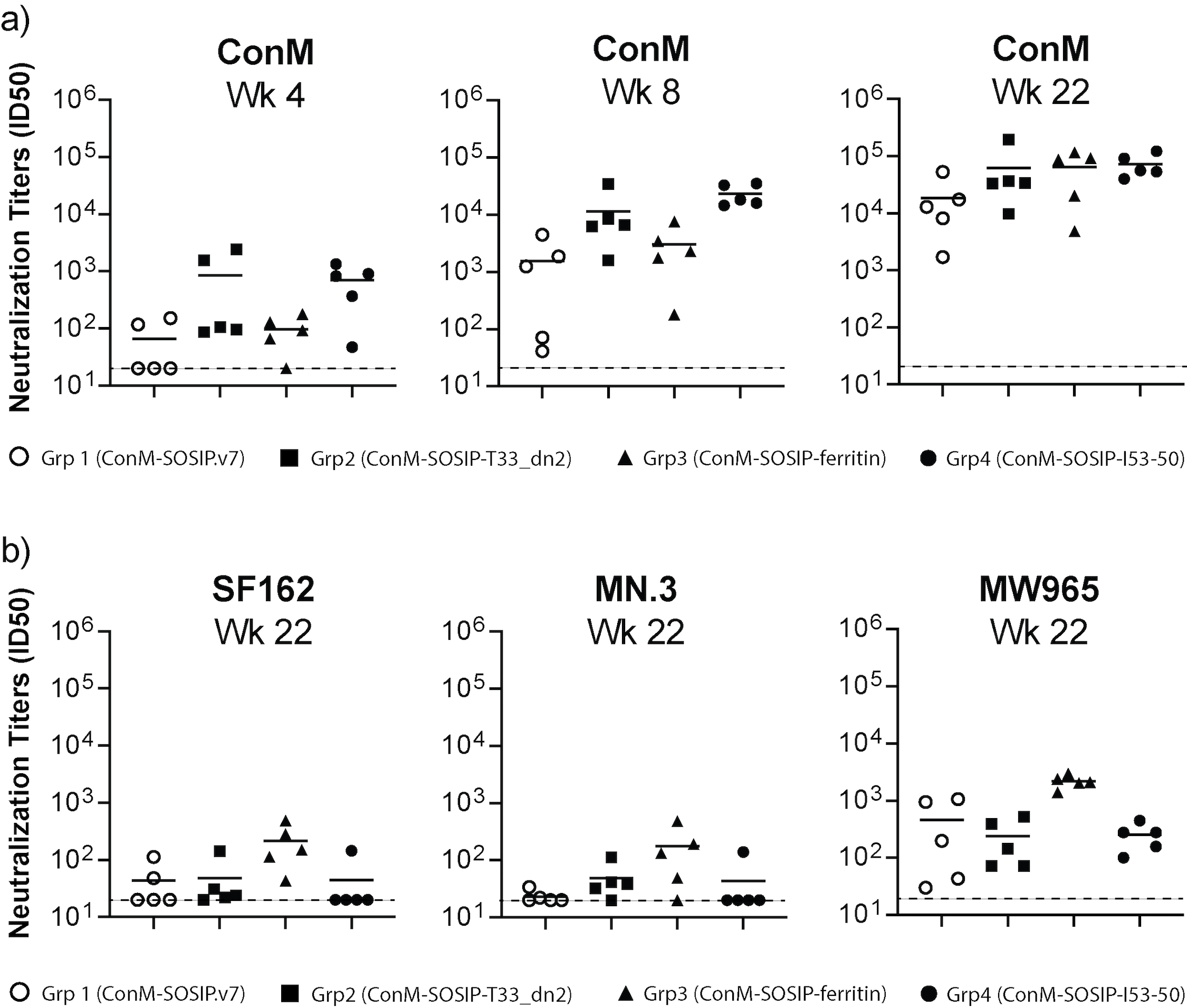

Supplement: S9 Fig — Neutralizing antibody titers (ID50) in each animal at different time points are presented as scatter plots with the mean titers indicated by lines. Data corresponding to ConM-SOSIP.v7 (open circles), ConM-SOSIP-ferritin (black triangles) and ConM-SOSIP-I53-50 (black circles) immunogens was adapted from Brouwer et al., 2019 [37]. Data depicted with black squares corresponds to ConM-SOSIP-T33_dn2 nanoparticle immunogen. (a) Autologous neutralization titers (ID50) against the pseudotyped ConM Env virus (sera samples from weeks 4, 8 and 22). (b) Neutralization titers (ID50) against the selected heterologous Tier-1 viruses (sera samples from week 22 time point). (TIF) [file ppat.1008665.s017.tif]

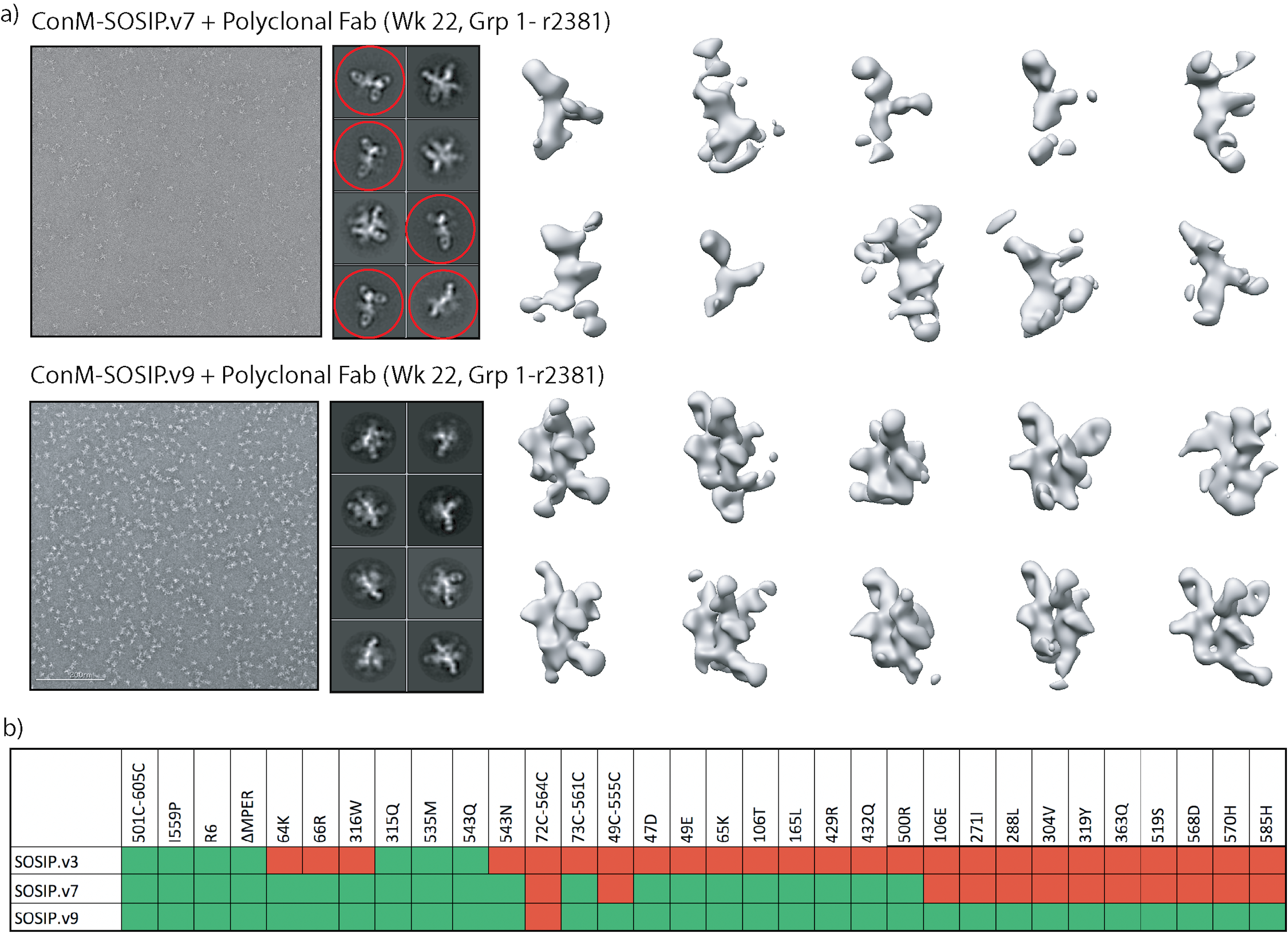

Supplement: S10 Fig — (a) EMPEM data derived using ConM-SOSIP.v7 (top) and ConM-SOSIP.v9 (bottom), complexed with polyclonal Fab sample isolated from the serum of rabbit r2381 (Grp1) at week 22 time point. Representative raw micrographs (left), 2D classes (middle) and 3D classes (right) are shown. Red circles mark the 2D classes of ConM-SOSIP.v7 gp120-gp41 monomers bound to one or more Fabs. Reconstructed monomer-like 3D classes are shown. Fabs are easily discernable in most 3D classes but epitope assignment is very challenging due to high degree of heterogeneity. Complexing with ConM-SOSIP.v9 results in significantly lower percentage of disassembled trimers (i.e. monomers), which can be observed in 2D and 3D classes. (b) Comparison of stabilizing mutations in different ConM-SOSIP constructs. (TIF) [file ppat.1008665.s018.tif]

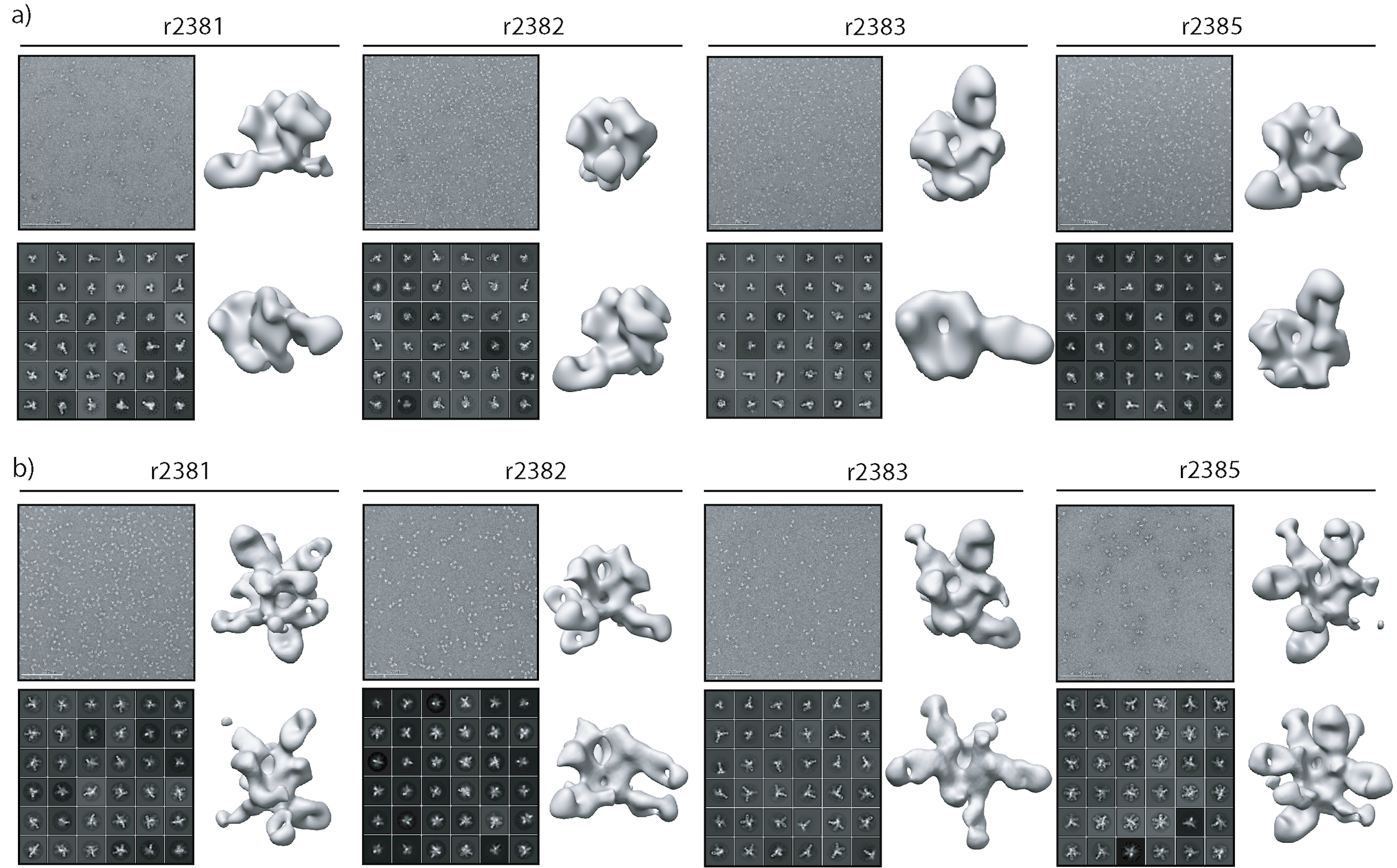

Supplement: S11 Fig — Sample micrograph, 2D class averages and sample 3D reconstructions obtained for antibodies isolated from the specified rabbits at (a) week 4 and (b) week 22. (TIF) [file ppat.1008665.s019.tif]
