# Supplementary material for: Structural and functional evaluation of de novo-designed, two-component nanoparticle carriers for HIV Env trimer immunogens
Source: PLoS Pathog. 2020 Aug 11;16(8):e1008665. doi: 10.1371/journal.ppat.1008665 (PMC7418955; doi:10.1371/journal.ppat.1008665)
Supplement: Attachment — Submitted filename: PLoS_Pathogens__Response_to_Reviewers_-_AA___05-15-20.docx [file ppat.1008665.s020.docx]

**Point by Point Responses to Reviewers’ Questions/Comments**

**Part I – Summary**

Please use this section to discuss strengths/weaknesses of study, novelty/significance, general execution and scholarship.

Reviewer #1: This well-written study seeks to address some of the drawbacks of soluble HIV Env trimer vaccine platforms – to generate nanoparticles that might enhance immunogenicity, e.g. by providing a multivalent array of trimers, helper epitopes, modified trafficking and/or blocking the hyperimmunogenic foot of these trimers. Composite immunogens were assembled from two components: SOSIP fused to an antigen-bearing component coupled with cognate, computationally designed assembly components. 5 initial formats were reduced to three that properly assemble for antigenic and structural analyses. Interestingly, the nanoparticle constucts showed an ability to stimulate nAb precursor expressing cell lines. One construct was then tested for immunogenicity in rabbits, where similar NAb titers were (slightly elevated) elicited as compared to soluble monomers. Overall, the work is high in concept, but a payoff yet remains unclear. One could compare this to a Prius (SOSIP) remodeled to swap the body with that of a race car (nanoparticle), which ultimately drives the same as the original Prius (immunogenicity) - though the reality may be somewhere in between.

A few specific comments follow:

It should be explained why the authors used a tier 1 ConM virus for immunogenicity? This might induce V3 responses that don’t inform vaccine design for efforts to develop products that might be broiadly effective against diverse circulating strains. In the mapping studies, it would be useful to do V3 peptide interference to determine how much this contributes to neutralization titers. It would be better to use BG505 SOSIP – a tier 2 trimer.

The choice of ConM as an immunogen was made for several reasons. The most important one is that the soluble ConM-SOSIP trimer is presently being tested in a human clinical trial (EAVI), so our results have a clinical context in the long term. It is true that the ConM virus, an artificial construct, has a Tier-1 neutralization phenotype. However, the soluble ConM-SOSIP trimers used in this study were engineered with SOSIP.v7 stabilizing mutations as described in Sliepen et al., 2019. The stabilizing mutations in ConM-SOSIP.v7 trimers have been demonstrated to effectively suppress the antigenicity of epitopes in V3 and the CD4i site that dominate the Tier-1 NAb response (see the antigenicity studies included here and elsewhere (Sliepen et al., 2019; Brouwer et al., 2019)). Another point is that the ConM-SOSIP.v7 trimers contain a full set of PNG sites, at least at the primary sequence level, and hence may lack large holes in the glycan shield that are immunogenic for narrow-specificity NAbs. The antigenicity profile of the ConM-SOSIP.v7 trimer, therefore, more closely reflects those of Tier-2 viruses. The immunogenicity of ConM-SOSIP.v7 trimers has already been evaluated in rabbit and non-human primate animal models (Sliepen et al., 2019). In these experiments the V3-directed antibody responses have been found to be minimal or non-detectable. Importantly, the immunogenicity of the ConM-SOSIP.v7 trimer on octahedral ferritin and icosahedral I53-50 nanoparticles presenting 8 and 20 copies of the trimeric antigen on the surface has been recently described (Brouwer et al., 2019). These studies and ours were contemporaneous and were carried out under the same conditions (animal model, dose, immunization regiment, adjuvant) to permit a comparison of data derived using immunogens of different valencies.

In order to address the reviewers concerns regarding the ConM-SOSIP immunogen used in this study, we have (1) included a more extensive clarification regarding the selection of ConM for the study (lines 365-377 in the revised manuscript text); (2) provided additional antigenicity data (S7d Fig, lines 378-404 in the revised manuscript text); (3) expanded the immunogenicity section with results from heterologous virus neutralization assays, including a panel of Tier-1 viruses (S7 and S8 Tables, lines 431-442 in the revised manuscript text); and (4) performed a comparison between the immunogenicity of different nanoparticle platforms presenting ConM-SOSIP.v7 (4, 8 and 20-valent) in the Results and Discussion sections (S9 Fig, lines 443-462 in the revised manuscript text).

Related to this, fig S7c, where are V3 mAbs binding data?

We have now provided additional SPR data, including experiments with immobilized antigens and mobile-phase monoclonal antibodies (S7d Fig). Among the tested monoclonal antibodies, 14e, 17b, 19b, 39F and PGT122 have epitopes comprising V3 amino-acid residues and N-linked glycans. All broadly-neutralizing antibody (bNAb) probes (including PGT122) showed strong binding to ConM-SOSIP.v7 (as soluble trimer and nanoparticle). The V3-targeting non-NAbs (14e, 17b, 19b and 39F) displayed no or minimal binding. The b6 non-NAb with specificity towards the CD4 binding site also showed no measurable binding to the same antigens. Altogether, the data suggest that the stabilized ConM-SOSIP antigens presented on the nanoparticle are well-folded, native-like trimers. This is further elaborated in the Results section (lines 378-404 in the revised manuscript text).

Also, what is MAb 8D9??

We apologize for the confusion regarding the nomenclature of 8D9 antibody. It was the original name used for the fusion-peptide-directed antibody RM20F described by Cottrell et al., 2020. We have now corrected the error.

35022 binding seems not to be reduced against particles.

In the SPR experiments with immobilized monoclonal antibodies and antigens in the mobile phase (S7c Fig) the binding signal is a function of affinity as well as of the molecular mass of the bound antigen. The stronger binding signal in experiments with the apex and CD4bs-directed antibodies is detected because the molecular mass of the ConM-SOSIP T33_dn2 nanoparticle is ~5.1 times higher than that of ConM-SOSIP trimer. Antibodies with epitopes closer to the trimer base (including 35O22) yield comparable binding signals between the trimer and nanoparticle antigens. However, due to the molecular mass differences, this result suggests that there is a relatively decreased affinity towards the nanoparticle antigen. This finding is consistent with the data collected using BG505-SOSIP-displaying nanoparticles reported elsewhere (Ueda, Antanasijevic et al., 2020; Brouwer et al., 2019). To clarify this point, we have included additional explanations in the Results section (lines 386-398 in the revised manuscript text).

We have also added SPR data derived using immobilized antigens and monoclonal antibodies in the mobile phase (the converse format to that originally used; S7d Fig, lines 398-404 in the revised manuscript text). The new data offer complementary information and is more straightforward to interpret (i.e. the binding signal is recorded while flowing monoclonal antibodies so there is no MW difference). In the experiments with 35O22, we observe a reduced binding signal with the nanoparticle antigen (ConM-SOSIP-T33_dn2) compared to free trimers (ConM-SOSIP.v7), consistent with the results of SPR experiments with immobilized mAbs.

On the serum mapping, the data implies glycan epitopes like N611, N625 etc, but are glycan contacts actually made or are these glycans just use to assign regions and the sera actually bind underlying protein? Since glycan holes are typically preferred, are there any in the ConM sequence and to what extent do NAbs target these holes? (i.e. check by hole filling, etc).

In the EMPEM experiments, the terms “N611”, “N618” and “N625” are used as short-hand to designate antibody epitopes on the surface of the trimer. The resolution of negative stain EM is sufficient to conclude that footprints of elicited polyclonal antibodies are partially overlapping with the potential N-linked glycosylation (PNG) sites. However, from NS-EM we cannot infer if the antibody interaction is dependent on the presence of the glycans at each site or if the binding is to a site that is unoccupied by a glycan. At the primary sequence level the ConM-SOSIP trimer contains all the PNG sites and hence lacks large holes in the glycan shield that may well be immunogenic (Sliepen et al., 2019). However, data on site-specific glycosylation in ConM-SOSIP trimers has not been published, so we do not have information on the percentage occupancy at each PNG site. From studies of the BG505-SOSIP trimer, we know that gp41 glycans (N611, N618, N625 and N637) can have occupancies as low as 25% (Cao et al., 2017; S3 Fig). However, in the case of BG505-SOSIP, the presence of the nanoparticle component at C terminus significantly improves occupancy at these sites (S3 Fig), which further complicates the comparative analysis of the elicited antibody responses.

To address the reviewer’s question we have added a paragraph discussing this topic to the results section (lines 478-486 in the revised manuscript text).

line 29: “fit the requirements of different immunization strategies” this implies that something is known about what immunization strategies might succeed. Is this known? Hence, it might be better to say to “test novel multimerization concepts in immunization strategies to potentially improve antibody precursor stimulation and neutralizing responses”.

We thank the reviewer for this comment, and have revised the sentence as follows: “Computational design of protein nanoparticles with differing sizes and geometries enables combination with antigens of choice to test novel multimerization concepts in immunization strategies where the goal is to improve the induction and maturation of neutralizing antibody lineages.”

In rabbits, the differences in NAb titers/specificities did not correlate well with the promise of the antigenic stimulation studies seen using cell lines. This may be because the epitopes targeted by rabbits are type specific (V3?) and differ from these prototype antibodies that may be harder to induce. It would be useful to cover this point.

This is a very interesting question and a very important one to address in the future (particularly when it comes to comparing the immunogenicity of Env trimers in animal models and human clinical trials). We believe that the off-target responses (against the trimer base and the nanoparticle core) were the main limiting factors in this immunization, and the main reason why the benefits of nanoparticle presentation (multivalent interactions, better B-cell stimulation, improved trafficking) were not fully reflected in a substantially increased neutralizing antibody response. Antibodies targeting the base of the trimer are very efficiently elicited in different animal models and are not specific to rabbits. Also, we found no evidence that V3 reactivity played a major role in these experiments. As noted above, the ConM-SOSIP.v7 trimer has been shown to antigenically resemble a Tier 2 virus and to elicit minimal anti-V3 antibody responses in rabbit and non-human primate animal models. Using EMPEM we found no detectable antibodies targeting the V3-tip. However, we observed several different antibody responses targeting non-V3 epitopes, which are likely to be neutralizing. In the revised manuscript we have also incorporated the results of pseudovirus neutralization assays with Wk22 sera against a panel of heterologous viruses (S7 and S8 Tables), including a subset of Tier 1a viruses (SF162, MN.3 and MW965). Minimal serum cross-reactivity was observed with SF162 and MN.3 viruses (IC50 values are in a ~10^1^-10^2^ range), while with MW965 the cross-reactivity was somewhat higher (IC50 values ~10^1^-10^3^). These values are significantly below the neutralization titers against the autologous virus (IC50 values are in a ~10^3^-10^5^ range), consistent with the non-V3 phenotype.

The neutralizing response in all rabbits was highly strain-specific. This is most likely influenced by the antigen itself and the location/exposure of neutralizing epitopes, but also, the rabbit antibody repertoire. More work is necessary to investigate if this response can be broadened.

We have now included broader discussion of the immunization results in the Discussion section (lines 546-604 in the revised manuscript text).

Checking the immunogenenicity of separate SOSIP+unassembled scaffolds together in rabbits would have been a better comparator than just isolated SOSIP (fig 5) to determine if the modest benefit in NAb titers might come from providing helper epitopes in co-immunization. This control would have been especially useful, considering the anti-base antibodies recovered in the nanoparticle group, suggesting disassembly, raising questions about whether the modest NAb differences may be related simply to the co-presence of the two components in the immunizations (rather than their assembled particle). For example, helper epitopes in the scaffolds co-immunized could account for the differences.

This is an excellent suggestion that we plan to address in the future. However, we believe that several different factors may contribute to the observed increase in NAb titers, in addition to a T-cell based mechanism. (1) B-cell stimulation experiments (Fig 4) demonstrate that assembled nanoparticles but not free trimers strongly activate antigen-specific B-cells in vitro. (2) While it remains possible that nanoparticles might have disassembled in vivo, BLI experiments show that base (and base-proximal) epitopes remain at least partially accessible even on assembled nanoparticles. Hence, antibody-responses against these epitopes may be elicited even when the nanoparticles remain intact (although perhaps to a lesser extent than with the same soluble trimers). (3) The trafficking and lymph node follicle accumulation of BG505-SOSIP trimer-presenting T33_dn2 nanoparticles is better than seen with the equivalent amount of free BG505-SOSIP trimers after primary immunization of immunologically naïve rhesus macaques (Martin, Cottrell, Antanasijevic et al., 2020). This early advantage in antigen trafficking may be what drives the observed initial increase in NAb titers. (4) Marcandalli et al., 2019 described the immunogenicity of prefusion RSV F DS-Cav1-presenting I53-50 nanoparticles. That study evaluated the immunogenicity of a “non-assembling control” immunogen comprising DS-Cav1 genetically fused to the nanoparticle trimer and a variant of the nanoparticle pentamer that is incapable of driving assembly. That control immunogen elicited binding and neutralizing antibody titers in mice indistinguishable from trimeric DS-Cav1, suggesting that T helper epitopes in the fused nanoparticle trimer (and non-interacting nanoparticle pentamer) did not contribute to the enhanced immunogenicity of the assembled nanoparticle immunogen. Instead, the data directly linked the more potent humoral response to formation of the nanoparticle structure. Furthermore, pre-immunization with the unliganded I53-50 nanoparticle scaffold followed by immunization with DS-Cav1-I53-50 did not improve the antibody response to the antigen compared to when the pre-immunization step was omitted. This outcome suggests that the recall of Th-cells specific to nanoparticle scaffold epitopes did not have a profound effect on the development of the antibody response.

The review of nanoparticle approaches and the concept to provide co-stimulation signals is good. However, what is not clear is what the mentioned “appropriate” antigenic spacing actually means and how these constructs meet those needs. The particle size, antigen spacing, flexibility, possible lateral movement of trimers (as possible in membranes) could be important to ensure crosslinking of more than one surface IgM on a precursor cell. Calcium flux experiments seemed to be supportive, but it is unclear if these had much bearing in immunogenicity experiments (as covered above).

As the reviewer pointed out, the reference to “appropriate antigenic spacing” was meant to imply that de novo nanoparticle platform is customizable and can accommodate the presentation of antigens in different geometries (orientation, density and spacing). By adjusting the length and amino-acid composition of the linker between the SOSIP and the antigen-bearing component, one can at least partially control the level of flexibility in the system (and the lateral movement of trimers). The overall goal is to identify what drives the optimal immune response against the antigen, which will likely be epitope-dependent and has to be empirically determined. We did not assess the effects of nanoparticle geometry on immunogenicity in this study but plan to do so in the future. We have clarified this point in the Introduction (lines 111-119 in the revised manuscript text), including by changing the wording at places to eliminate any confusion.

Related to the above, Line 104: re multivalent geometry and spacing “This may be particularly important for vaccine design efforts focusing on a specific set of HIV Env epitopes, which need to be presented in an appropriate and accessible orientation.” I am not sure what this sentence implies. What scenarios come to mind? What specific set of epitopes and how might the strategy be tailored?

The data from BLI and SPR experiments presented here and elsewhere (Ueda, Antanasijevic et al., 2020, Brouwer et al., 2019) suggest that nanoparticle diplay can affect the binding of antibodies to epitopes that are close to the base of the trimer. In the simplest format, we have envisioned that by increasing the density of Env trimers one can decrease the accessibility of epitopes near the base. In other words, nanoparticles with high antigen density may be better suited for immunization strategies focusing on the elicitation of antibodies against the trimer apex and nearby epitopes. However, if the focus is on the epitopes that are closer to the Env-trimer base (the fusion peptide, the 3BC315 and 35O22 epitopes), then nanoparticles with greater antigen spacing (e.g., by lowering the maximum valency) must be designed to make sure that such epitopes are in fact accessible. Furthermore, by using this platform we can assess if modest changes in valency, particle diameter, antigen spacing and linker flexibility can significantly improve various aspects of the immune response. The I53-50 nanoparticle system for presenting DS-Cav1 (from RSV) at different densities has already been used in this way (Marcandalli et al., 2019). We have now further discussed these points in the Introduction (lines 111-119 in the revised manuscript text).

Line 40: The statement “increased the proportion of the overall antibody response directed against autologous neutralizing Ab epitopes present on the ConM-SOSIP trimers” is a bit ambiguous. A better concluding sentence in the introduction could be something like “Although neutralizing antibody responses exhibited a similar (slightly elevated) frequency titer and breadth (with a greater initial apex focus)”.

We have modified the concluding sentence of the Abstract as follows: “When the immunogenicity of ConM-SOSIP trimers presented on a two-component tetrahedral nanoparticle or as soluble proteins were compared in rabbits, the two immunogens elicited similar serum antibody binding titers against the trimer component. Neutralizing antibody titers were slightly elevated in the animals given the nanoparticle immunogen and were initially more focused on the trimer apex. Altogether, our findings indicate that tetrahedral nanoparticles can be successfully applied for presentation of HIV Env trimer immunogens; however, the optimal implementation to different immunization strategies remains to be determined.”

Line 53: How can immunogenicity in vitro = precursor cell stimulation? In vitro immunogenicity seems a contradictory term and could be reworded. There should be something straightforward on whether NAb responses were affected, as in the abstract.

We thank the reviewer for the comment. The problematic sentence has been modified to:

“The designed nanoparticle immunogens had an increased capacity to stimulate B-cells expressing antigen-specific receptors. The immunogenicity of one nanoparticle candidate was assessed in rabbits. Nanoparticle presentation geometry appeared to alter the distribution of antibody responses against different epitopes while inducing similar serum binding titers and only slightly elevated neutralizing titers.

Line 94: “precisely defined”… seems less jarring/repetitive sub with “well-defined”

We have modified the problematic sentence to now read: “Two-component, self-assembling nanoparticles are a versatile platform that can present HIV Env and other viral glycoproteins in a well-defined manner.”

The authors could briefly mention more about the success or not of various nanoparticle approaches in the paragraph at line 107. So far, have they been any better or not? Equivalent? Promising? Variable? Where are the knowledge gaps? The statement that they “provide additional (vaccine/antigen) options” (line 111) is a bit vague, given that there are published studies that should by now give some idea whether these options are actually useful.

To address the reviewer’s suggestions, we have now added to the Introduction a paragraph discussing the immunogenicity data derived from other nanoparticle platforms (lines 123-136 in the revised manuscript text).

S1 Fig/Table 1 Clarity question: why are assembly components in blue sometimes component A for tetrahedral scaffolds and other times component B? I don’t see why they should be switched? I am not sure if the nomenclature of the various antigen bearing and assembly components needs to be explained somehow better. As it stands it is confusing so an explanation of what A and B means and the other parts of name would help maybe as a brief nomenclature or label the components. I don’t know what the 33, 43 the dn means in T33 O43_dn18

We apologize for the confusion. The nomenclature originates from the design step where the nanoparticle component that has higher symmetry (e.g. 4-fold vs 3-fold in O43_dn18 nanoparticle) is termed component A while the lower-symmetry oligomeric building block is termed component B. For tetrahedral nanoparticles where both components are trimers, the naming is determined based on the origin of the building block. This system is explained in the paper by Ueda, Antanasijevic et al., 2020 that describes the *de novo* design of the two-component nanoparticles we have evaluated here. For clarity, we have now added an explanation on the naming system used (S1c Fig).

Although the v5.2 mutations and MD39 ones may be published, I would suggest they be listed here or highlighted in the sequence in the supp as it should take little space and will save the less committed but curious reader from needing to access other papers and makes this paper more self-contained in the process. It is curious that 14e is not exposed here (Fig 1) whereas it is highly exposed in other preps of SOSIP (e.g. Sanders JBC paper 2019). Whether this is due to an aromatic residue masking the V3 tip as reported previously or a genuine occlusion of the V3 is not clear on the face of the information accessible in this paper. As mentioned above, the V3 exposure on the ConM SOSIP is missing.

We agree that our use of different SOSIP versions and engineered stabilizing mutations can be confusing. We have now added a Table with the information on stabilizing mutations in SOSIPv5.2, MD39 and SOSIPv5.2(7S) constructs to the Supplement (S2 Table). The BG505-SOSIPv5.2(7S) construct is engineered to include the A316W mutation (from SOSIPv5.2) as well as the A319Y and R304V mutations (from MD39). The goal of these changes is to stabilize the V3 tip. We detect significantly a lower percentage of “open” trimer conformations for this construct, as shown by the poor reactivity of selected non-NAbs. The BLI experiments were performed with 14e (included in the paper, Fig 1 and Fig 2) and 19b V3-directed antibodies (included below and in the revised version of the manuscript text; S2 Fig). The soluble and nanoparticle trimers reacted minimally with these antibodies. However, as the reviewer pointed out, we cannot exclude the possibility that the lack of 14e and 19b reactivity is influenced by the stabilizing mutations that are located within the epitope. To further assess the trimer homogeneity, the BLI experiments were performed with non-NAbs that target the CD4 binding site, b6 (included in the paper, Fig 1 and Fig 2) and F105 (included below and in the revised version of the manuscript text; S2 Fig). The lack of binding to these antibodies also suggests that the BG505-SOSIP trimers are stabilized in the native-like pre-fusion conformation, with only a minimal proportion of open trimers present (if any). Negative-stain-EM analysis of the purified trimers (Fig 1) further supports these findings.

The V3 reactivity data for ConM-SOSIP.v7 trimers and ConM-SOSIP-T33_dn2 nanoparticles is now included in S7 Fig and the Results section (lines 398-404 in the revised manuscript text). In the SPR experiments, ConM-SOSIP trimers did not bind the 14e, 17b, 19b, 39F or b6 non-NAbs when they were presented on the T33_dn2 nanoparticle.

Figure: BLI experiments with 19b and F105 antibodies (data not included in the original version of the manuscript)

Line 201: not dn2A, but dn5B in Fig S3 (all mannose)

We apologize for the confusion. There was an error in the text (dn2A and dn5B were swapped). It has now been corrected.

Fig 4 colors are not very distinct, too dull to discern types

The panels in Fig 4 are now bigger and the line colors are changed to improve clarity.

The comprehensive listing of their SOSIP human clinical trials in the introduction is not necessary to repeat in discussion.

We have removed the Clinical Trials references from the Discussion.

Reviewer #2: In this manuscript by Antanasijevic et al., the authors characterize multimeric nanoparticle immunogens derived from BG505 and ConM SOSIPs, using a two-component, self-assembling, nanoparticle platform. The authors designed and characterized a nanoparticle library consisting of five different constructs based on BG505 SOSIP.v5.2(7S): three tetrahedral (T33_dn2, T33_dn5, T33_dn10), one octahedral (O43_dn18), and one icosahedral (I53_dn5). Of the five constructs, O43_dn18 did not express and T33_dn5 did not assemble efficiently. The three remaining antigen-bearing components were characterized for antigenicity through antibody binding, negative stain electron microscopy (only T33_dn10 and I53_dn5), and glycan profiling and were found to be similar to the parental BG505 SOSIP in key parameters. The T33_dn2 construct was somewhat surprisingly recognized to similar levels by base binding antibodies as the free SOSIP. When the antigen-bearing components were assembled, I53_dn5 did not assemble efficiently. However, all of the constructs had better activation of B cells expressing PGT145, VRC01, and PGT121 BCRs compared to wildtype, although T33_dn2 was the least efficient in all three cases. Nevertheless, the T33_dn2 construct was selected as the platform to display a different SOSIP, CON-M, for immunogenicity testing in rabbits. The free CON-M SOSIP and T33_dn2 immunogens elicited similar serum binding antibody and neutralizing antibody levels, and antibody specificities detected by negative stain EM mapping were also similar in the subset of animals analyzed, including the presence of base binding antibodies.

Overall, the paper is technical in nature and the majority of the paper is devoted to describing the generation and characterization of a panel of multimeric SOSIP antigens. The rationales for moving forward with the T33_dn2 platform and for switching from BG505 to the less characterized/optimized CON-M multimer were not clear. Since the T33_dn2 CON-M immunogen produced binding and neutralizing antibody titers that were very similar to the free trimer, there is no significant advance in immunogen design provided nor is there a compelling reason presented to pursue this approach that would be of interest to a broad readership.

We respectfully disagree with the reviewer and think that multiple valuable lessons were learned over the course of this study. Notably, we described in great detail the incorporation of two different Env trimers into self-assembling nanoparticles and methods to evaluate them structurally and antigenically. The focused refinement approaches used for high-resolution structural characterization of flexibly bound antigens, comparison of Env-presenting nanoparticles of different geometries, and the nanoparticle stability assays are first described in this work. We also assessed the immunogenicity of a well-behaved nanoparticle candidate T33_dn2. This nanoparticle can be expressed at high levels and has excellent stability; its low valency (4 trimers per particle) makes it a valuable reagent for studying the impact of nanoparticle size and trimer density on different aspects of the immune response. ConM is an Envelope sequence that has been widely used in the field and ConM-SOSIP trimer is now in human clinical trials (EAVI). This sequence is also the basis of an evaluation of octahedral ferritin and icosahedral I53-50 nanoparticles in rabbits (Brouwer et al., 2019). That study allows for a direct comparison with the immunogenicity data on the tetrahedral nanoparticle system in this study (included in the updated text), to thereby define guidelines for further nanoparticle immunogen design. Based on the results presented here we have already started modifying both the nanoparticle and Env designs to improve immunogenicity, something that we and many others in the field regard as an iterative process. We believe there is value in reporting results both positive and negative, so that others can learn from our work as we collectively lay a foundation for future work with self-assembling NPs that are now widely regarded as an important vaccine development platform.

Reviewer #3: Antanasijevic, Ueda et al brings together a number of tools – recombinant, native-like HIV-1 Env trimers and two-component, self-assembling nanoparticles – to generate multivalent vaccine candidates. The general design and structural characterization of these kinds of nanoparticles are described in a bioRxiv paper by the same group (Ref. 38, Ueda, Antanasijevic et al). This manuscript focuses on detailed characterization of the class of particles that display HIV-1 Env spikes. Initial studies were done on the BG505-SOSIP trimer, and then the authors switched to consensus group M (ConM)-SOSIP trimers. Expression and assembly data on various test constructs, BLI binding experiments to test antigenicity, and glycan composition analyses are shown. CyoEM structures of tetrahedral and icosahedral particle types were determined, which confirm the expected structures of both the nanoparticle module and trimer modules. The capacity of the nanoparticles to stimulate antigen-specific B cells was evaluated; it was shown that assembled nanoparticles activated the B cells more strongly, compared to the control (free trimers). Finally, immunogenicity of the ConM-SOSIP nanoparticles was determined in rabbits.

In general, the overall study seems well-designed and executed, and the manuscript presents it in a logical and comprehensible manner. In vitro antigenicity profiles were only done in a qualitative manner (no affinities were determined by BLI), yet the qualitative data are sufficient to show that the nanoparticle design left much of the spike available for presentation and identify occluded regions. The structural analysis seems solid. If the goal of the authors is to present these nanoparticles as viable vaccine candidates then they have succeeded; in this regard the impact and interest of the manuscript is primarily as a proof-of-principle study that may end up occupying a prominent position among the many similar such studies available in the literature. In this reviewer’s opinion, it is primarily a technical paper (with the most interesting aspects already described in the bioRXiv paper, the final form of which will be presumably published elsewhere); thus it falls somewhat short of the kinds of mechanistic and biological insights that one might expect from a paper in PLoS Pathogens.

We thank the reviewer for a very insightful summary. We agree that the paper was written in a manner that stresses the analysis of nanoparticles, and for that reason it may appear “technical” in nature. The main idea behind the study was to comprehensively evaluate different aspects of production, assembly, stability, structure, antigenicity and immunogenicity of the *de novo* designed nanoparticles and to present them objectively. Additionally, some of these methods had to be established (e.g. nanoparticle assembly and stability experiments) or adapted (e.g. localized reconstruction method for structural studies) for the analysis of nanoparticles and in this format they are first described in this work. However, the paper offers more than just technical details on the designed antigens and provides valuable insights for vaccine design and optimization. For example, using site-specific glycan analysis we show that addition of extra domains at the C terminus of BG505-SOSIP can alter the glycosylation profile of the trimer and increase occupancy at the gp41 sites. This can be used to immuno-silence the potential glycan holes caused by partial glycosylation at these sites and increase the homogeneity of nanoparticle-presented trimers. Also, we describe two well-behaved tetrahedral nanoparticle candidates presenting 4 trimers on the surface and demonstrate that, despite the low valency, these nanoparticles can effectively stimulate B-cells that are specific to the antigen. Rabbit immunization studies in combination with EMPEM provide valuable data for future immunization efforts with nanoparticles, but also with ConM trimers, both being important reagents in the HIV vaccine design field.

In order to address the reviewer’s comments and incorporate more biological and mechanistic data we have now included additional immunization data and more general discussion of the results of our *in vivo* studies (presented here and elsewhere). These include:

1) Cross-neutralization data using heterologous viruses and sera samples from trimer and nanoparticle-immunized groups of rabbits (S7 and S8 Tables, lines 431-442 in the revised manuscript text).
2) The immunogenicity of ConM-SOSIP.v7 trimers in the context of octahedral (ferritin) and icosahedral (two-component) nanoparticles has already been evaluated and published in the manuscript by Brouwer et al., 2019. This study was performed in parallel to the immunization experiments presented here and under equivalent conditions (animal model, adjuvant, immunogen dose and immunization regimen). We have now included data comparing nanoparticles of different geometries and valencies (tetrahedral, octahedral and icosahedral) in the Results section (S9 Fig, lines 443-462 in the revised manuscript text).
3) Recently we have completed a study dealing with trafficking of trimer and tetrahedral nanoparticle immunogens (using T33_dn2 system) in non-human primates (Martin, Cottrell, Antanasijevic et al., 2020). In the revised manuscript we have now included a section to discuss the trafficking data in the context of this paper (primarily the immunization data), while we attempt to dissect different factors contributing to the immunogenicity of the presented HIV Env trimers and the development of antibody response (see updated Discussion Section in the revised manuscript text).

We believe that the revised manuscript that now includes the abovementioned edits will have more conclusions of relevance for the researchers focusing on vaccine design as well as a more general readership.

**Part II – Major Issues: Key Experiments Required for Acceptance**

Please use this section to detail the key new experiments or modifications of existing experiments that should be absolutely required to validate study conclusions. Generally, there should be no more than 3 such required experiments or major modifications for a "Major Revision" recommendation. If more than 3 experiments are necessary to validate the study conclusions, then you are encouraged to recommend "Reject".

Reviewer #1: (No Response)

Reviewer #2:

1. The authors should provide a stronger rationale of why BG505 SOSIP was used for structural and in vitro functional characterization and optimization studies but ConM SOSIP was used in the in vivo studies.

Both BG505-SOSIP and ConM-SOSIP trimers are being evaluated in clinical trials and are both highly relevant reagents. BG505-SOSIP was considered for this study, but was replaced with ConM for several reasons. (1) We wanted to evaluate if the *de novo* nanoparticle platform can be used with other HIV Env trimer sequences. (2) ConM-SOSIP trimers have been found to elicit strong neutralizing antibody response against the V1/V2 loops at the trimer apex (Sliepen et al., 2019). Despite the fact that this response is primarily autologous, it provides an excellent readout for comparison of different platforms for immunogen presentation and/or different immunization strategies. (3) The timing of the study was aligned with the immunization experiments with ConM-SOSIP trimers and nanoparticles described in Brouwer et al., 2019. The tetrahedral nanoparticle, T33_dn2, presenting ConM-SOSIP, was evaluated in these immunization experiments alongside ferritin (octahedral) and I53-50 (icosahedral) nanoparticles, allowing for direct comparison with these well-established nanoparticle platforms.

In the revised manuscript text we have included a more extensive explanation regarding the usage of ConM (lines 365-377 in the revised manuscript text). Also, we have added the heterologous neutralization data and made the comparison to the ferritin and I53-50 nanoparticles (adapted from Brouwer et al., 2019) (S9 Fig, lines 443-462 in the revised manuscript text).

2. The authors characterized the antigen-bearing component using a panel of Nabs and two non-Nabs, RM19R and RM20A3. These non-Nabs were included because they are base binding antibodies that are not expected to bind to the antigen-bearing component because this is where the antigen fuses to the nanoparticle. Two of the antigen-bearing components did not bind these antibodies but T33_dn2A showed binding similar to free BG505-SOSIP.v5.2(7S). The authors need to elaborate on why this occurred and support their selection of this construct to move forward.

The underlying helical scaffold that was used for fusion to BG505-SOSIP in T33_dn2A (trimeric scaffold: 1na0C3_3; (Fallas, Ueda, Sheffler et al., 2017)) was different from the T33_dn10A and I53_dn5B components, which are highly similar to each other (trimeric scaffold: 1na0C3_2; (Ueda, Antanasijevic et al., 2020)). These constructs also utilize different linkers to connect the scaffold to the C terminus of BG505-SOSIP trimers. This strategy affects the expression properties of different antigen-bearing constructs. For example, SOSIP-T33_dn2A fusion constructs are less prone to nanoparticle-scaffold-mediated self-aggregation (S2 Fig) and they can be produced at higher levels than the SOSIP-T33_dn10A and SOSIP-I53_dn5B fusions. Additionally, T33_dn2 nanoparticle has excellent assembly properties and stability, particularly when compared to the I53_dn5 nanoparticle. These factors in combination with the extensive experience we have gained on the T33_dn2 system, were the main motivators to use this nanoparticle platform for the immunization experiments.

The differences in scaffolds and linkers used in SOSIP-T33_dn2A fusion constructs on the one hand and SOSIP-I53_dn5B and -T33_dn10A on the other may impact how they interact with the base-binding antibodies. Both of these antibodies directly interact with the C-terminal helix in the gp41 subunit of the BG505-SOSIP trimer, which makes the exact comparison of binding properties problematic. A larger (but not yet available) panel of base-binding antibodies covering different epitopes and angles of approach will be necessary to truly evaluate/compare the accessibility of the trimer base in different constructs/nanoparticles. Only then could predictions be made as to whether immunogenicity might be improved.

We have included additional explanations to the Results section to (1) elaborate on the observed difference in base accessibility in different antigen-bearing components (lines 217-220 in the revised manuscript text) and (2) the choice of this nanoparticle for immunogenicity studies (lines 352-364 in the revised manuscript text).

3. The authors fail to address why cryo-EM was not performed on BG505-SOSIP-T33_dn2A. This is especially important because this platform was chosen to move forward in the rabbit studies.

The cryo-EM analysis on BG505-SOSIP-presenting T33_dn2 nanoparticle has been performed and the data will be presented elsewhere (Martin, Cottrell, Antanasijevic et al., 2020, in revision at NPJ Vaccines). We have included the necessary clarification and references in the Results section (lines 291-292 in the revised manuscript text). In the same paper, we described the trafficking of nanoparticle-presented trimers to the lymph nodes of Rhesus macaques and demonstrated that, despite the modest valency of 4 (trimers per nanoparticle), the presentation of SOSIP trimers on T33_dn2 particles resulted in significantly increased localization of antigen into the lymph node follicles. These data were another reason why the T33_dn2 nanoparticle platform was selected for the rabbit immunization experiments. While we agree that it would have been useful to also include the BG505-SOSIP-T33_dn2 cryo-EM structure here, because of the earlier timing of the trafficking study the structure was included in that manuscript as part of the validation of the integrity and quality of the immunogen tested.

4. The authors point out a difference in early targeting of the V2 apex in the T33_dn2 immunized rabbits. However, only 2 animals from each group were analyzed by NSEM, and all animals had these antibodies by wk 22, so this conclusion is not supported by the data presented.

In this paper, we chose the two animals per group with the highest neutralization titers to profile by EMPEM. Because all animals eventually developed a V2-apex response but only the NP group developed it early, this finding correlates well with the autologous neutralization data. This inference is supported by the results described in Sliepen et al., 2019 and Brouwer et al., 2019, where the authors identify variable loops at the ConM trimer apex as the main epitope target for autologous neutralizing antibodies. As described in the Methods and Results sections of this manuscript, the immunization experiments described in Brouwer et al., 2019 and here were performed simultaneously and via a shared protocol; the control group of rabbits, receiving soluble ConM-SOSIP trimers (Group 1) was shared between the two studies, so the findings obtained for this group of animals are directly applicable here. Furthermore, in an unpublished nanoparticle experiment involving groups of 6 macaques, we have analyzed all the animals by EMPEM and observed the same consistent trend of an early biased apex response in animals immunized with Env-presenting nanoparticles (EMPEM results are shown below). Thus, we are confident that the trend we describe here will hold.

Because we are limited in the laboratory work we can perform on this HIV project during the present COVID-19 crisis, we cannot perform new experiments for the current manuscript. We have therefore modified the text to soften our conclusion (lines 469-470 in the revised manuscript), and request the editor’s understanding of the position we are in.

Figure: A) NHP immunization information and regimen; B) EMPEM data presented as composite figures; C) Summary of EMPEM data at different time points presented as bar-graphs

5. In Fig. S3, the text does not match the numbers shown for some of the glycan differences (ex. N637, N355), line 199. It appears that dn2A and dn5B could have been inadvertently switched.

We apologize for the confusion and thank the reviewer for pointing out the problem. There was an error in the text (dn2A and dn5B were switched) that we have now corrected.

6. The 8D9 and 3BC315 antibodies bound less efficiently to the CON-M SOSIP T33_dn2 than to the free trimer but 35022 bound better, (Fig. S7) which is not what is stated in the

text on line 336.

In these experiments, monoclonal antibodies were immobilized and antigens were passed over them in the mobile phase to assess binding (S7c Fig). However, in SPR the binding signal is a function of affinity as well as of the molecular mass of the bound antigen. The molecular mass of the ConM-SOSIP trimer is ~5.1 times lower than that of ConM-SOSIP-T33_dn2 nanoparticle, which is reflected in the binding signals detected with antibodies targeting epitopes at the trimer apex and CD4bs. Antibodies with epitopes at the gp120/gp41 interface (35O22), the fusion peptide (ACS202, PGT151, VRC34, RM20F) and interprotomeric gp41/gp41 interface (3BC315) show similar binding signals between the trimer and nanoparticle antigens. As the reviewer points out, the signal for 35O22 binding is higher for the nanoparticle than the trimer. However, when the molecular mass difference was included in the analysis, we concluded that the relative nanoparticle to trimer binding to 35O22 was reduced compared with the binding to the epitopes higher up the trimer. This conclusion is also supported by the SPR experiments with immobilized antigens where antibodies are a part of the mobile phase. Given that, in this setting, there was no molecular mass effect, the binding signals can be directly compared. These new results are included in the revised manuscript (S7d Fig). Additional explanations regarding the interpretation of the SPR data are also included in Results section (lines 386-404 in the revised manuscript text).

7. There is no negative control included in the neutralization assays. Given such high autologous titers, it seems that a negative control virus and purification of IgG would be prudent.

Serum neutralization of the pseudotyped MLV virus was also measured at weeks 4,6 and 22 as a negative control following each immunization. The ID_50_ titer values against MLV are now included in the S6 Table. MLV neutralization was mainly below the level of detection (ID_50_ < 20), except in a few cases where weak neutralization (20 < ID_50_ < 75) was detected. Given that the negative control experiments with MLV were not indicative of any problems with non-specific cross-reactivity we decided not to perform experiments with purified IgGs.

8. Given the use of CON-M SOSIP, was neutralization breadth measured? Presumably that is a reason for switching to CON-M.

Neutralization breadth was assessed by testing week 22 serum from all the animals against 9 pseudotyped viral strains from the HIV Env global panel. None of the serum samples (from the trimer and the nanoparticle group) cross-neutralized any of the tested HIV-1 strains. These data are now included (S7 Table) and discussed. Additionally, week 22 serum neutralization was measured against the ConS pseudotyped virus (a related consensus sequence virus, but one with a Tier 1b phenotype). Cross-neutralization of ConS was detected for serum samples from the soluble trimer and nanoparticle trimer groups. These data are also included (S8 Table) and discussed in the updated manuscript text (lines 431-442 in the revised manuscript text).

9. Given the desire to elicit differences in recognition of neutralizing antibody epitopes, envelope mutants should be used to determine whether there were in fact any distinctions between the free and T33_dn2 immunogens. This could shed light on whether the V2 antibodies seen in the NSEM analysis made any contributions to neutralization.

We thank the reviewer for the suggestion. As mentioned above, similar experiments have already been performed for the animals in the ConM-SOSIP trimer group (Group 1) and published elsewhere (Brouwer et al., 2019). Using competition ELISA and virus neutralization assays with serum depletion, the authors demonstrated that the epitopes at the ConM-SOSIP apex are the main target for neutralizing antibodies in rabbits from the ConM-SOSIP trimer group (as well as the group of animals receiving the ConM-SOSIP-I53-50 nanoparticle immunogens). This conclusion is also concordant with the autologous nature of the response, and the lack of breadth. However, we have not acquired corresponding data for the Group 2 of rabbits (immunized with ConM-SOSIP-T33_dn2 nanoparticle). While we agree that this would be a useful data set to see if the conclusions drawn in the Brouwer et al. 2019 paper are more generally true, we cannot perform the necessary experiments due to the COVID-19 imposed restrictions on working on this project in our laboratories.

Reviewer #3: (No Response)

**Part III – Minor Issues: Editorial and Data Presentation Modifications**

Please use this section for editorial suggestions as well as relatively minor modifications of existing data that would enhance clarity.

Reviewer #1: (No Response)

Reviewer #2: (No Response)

Reviewer #3: (No Response)
